# Assessing Structural Classification Using AlphaFold2 Models Through ECOD‐Based Comparative Analysis

**DOI:** 10.1002/prot.26828

**Published:** 2025-04-19

**Authors:** Takeshi Kawabata, Kengo Kinoshita

**Affiliations:** ^1^ Graduate School of Information Sciences Tohoku University Sendai Japan

**Keywords:** AlphaFold2, homology, protein structural comparison

## Abstract

Identifying homologous proteins is a fundamental task in structural bioinformatics. While AlphaFold2 has revolutionized protein structure prediction, the extent to which structure comparison of its models can reliably detect homologs remains unclear. In this study, we evaluate the feasibility of homology detection using AlphaFold2‐predicted structures through structural comparisons. We considered the classification of the ECOD database for experimental structures as the correct standard and obtained their corresponding predicted models from AlphaFoldDB. To ensure blind assessment, we divided the structures into test and train sets according to their release date. Predicted and experimental 3D structures in the test and train sets were compared using 3D structure comparisons (MATRAS, Dali, and Foldseek) and sequence comparisons (BLAST and HHsearch). The results were evaluated based on the homology annotations in the ECOD database. For top‐1 accuracy, the performance of structural comparisons was comparable to that of HHsearch. However, when considering metrics that included all structural pairs, including more remote homology, structural comparisons outperformed HHsearch. No significant differences were observed between comparisons of experimental versus experimental, predicted versus experimental, and predicted versus predicted structures with pLDDT (prediction confidence) values greater than 60. We also demonstrate that predicted protein structures, determined by NMR, had lower pLDDT values and contained fewer coils than their experimental counterparts. These findings highlight the potential of AlphaFold2 models in structural classification and suggest that 3D structural searches should be conducted not only against the PDB but also against AlphaFoldDB to identify more potential homologs.

## 1 Introduction

Homology is the similarity caused by shared ancestry in protein history and provides the basis for the natural classification of proteins [1]. Homology detection reveals the evolutionary history of proteins and their functional and structural relationships because homologous proteins often share many properties, such as molecular function, tertiary and quaternary structures, and binding molecules, during their evolutionary history [2, 3]. The group of proteins sharing homology is often called a “family” or “superfamily.” Statistically significant sequence similarity is regarded as solid evidence of homology; however, the sensitivity of sequence analysis is limited. Similarities in protein 3D structures have frequently been exploited to detect remote homology. This is based on the observations that tertiary protein structures are more conserved than their sequences in their evolutionary history [4]. Several databases for protein structure classification have been developed, including SCOP [5, 6, 7], CATH [8, 9], and ECOD [10]. These databases comprise structural domains, not the entire protein chains, and employ a hierarchical classification. Some structural regularities and symmetries arise from the intrinsic chemical and physical properties of proteins, even among nonhomologous proteins [11, 12]. The similarity from no evolutionary origins is called “analogy.” The databases have a classification level to distinguish homology and analogy: SCOP uses the “fold” for analogy and “superfamily” for homology, CATH employs “architecture” and “topology” for analogy and “homologous superfamily” for homology, and ECOD uses “architecture” for analogy, “X” for possible homology, and “H” for homology level. The discrimination between homology and analogy is controversial in some cases; human experts must determine the problematic cases [5, 13, 14, 15, 16]. Therefore, these databases employ semi‐automatic procedures for classification. In ECOD, standard sequence search methods (such as BLAST [17]) are first used to detect close homology between a query protein sequence and a sequence library of classified proteins. Then, if BLAST fails to identify any significant similarities, the profile‐profile comparison methods such as HHsearch [18] are performed to check for further remote homology. Then, three‐dimensional (3D) structure comparison methods, such as the program Dali [19, 20], are used to confirm and seek distant homology. If the detected similarities are weak and controversial, human experts determine the classification and occasionally manually change the domain partitioning [10].

3D structural data helps detect remote homology; however, the amount of available experimental 3D structure data is currently much lower than that of known protein sequences. Several studies have used predicted secondary structures [21, 22] and contact maps [23] instead of experimental structures. Predicted structures have been used to predict protein functions [24]. Recently, DeepMind proposed a highly accurate prediction method, AlphaFold2 (AF2) [25]. Following the remarkable success in CASP14 [26], the predicted models for over 350 000 proteins from 21 model organism proteomes were released as AlphaFoldDB (AFDB) with the assistance of the EMBL‐EBI [27, 28]. The database has recently expanded to additional models, including over 200 million protein structure predictions [29]. Many attempts to use AF2 have been proposed, particularly for structural modeling using CryoEM and protein designing. It has also been used for protein function prediction [30] and modeling of complex structures with ligands and co‐factors [31]. Structural comparison studies of AF2 models have also been reported. Akdel et al. used a fast comparison program to perform an all‐vs‐all structural comparison between AF2 and PDB structures [32]. They reported that clusters mainly composed of AF2 structures correspond to specific superfamilies and have unique structural features rarely seen in the PDB. Bordin et al. reported the classification of AF2 models into CATH superfamilies using their new classification protocol, CATH‐Assign [33]. They claimed that 73% of the AF2 models were classified using sequence analysis (CATH‐HMM), 8% were classified using the CATHe predictor based on protein language model [34] with validation by structure comparison, and 10% were classified only by structural comparison. The new protocol CATH‐Align, proposed by Bordin et al. in 2023, has been further refined and adapted as CATH‐AlphaFlow, which has been released as the latest CATH database [35]. Similarly, Schaeffer et al. classified AF2models of thehuman proteome into ECOD [36]. They reported that 47 566 human ECOD domains were assigned using their DPAM (Domain Parser for AlphaFold Model) [37]; and 23% (10 994 domains) were not modeled in the SWISS‐MODEL repository [38]. Their automatic pipeline classified 98% of the 47 566 domains; 65% (31 046 domains) could be classified among known folds by sequence, and 33% required structural data to refine domain boundaries or support homology. Schaeffer et al. have used this new DPAM protocol for the structural classification of PDB and AFDB and have released the results as the latest ECOD database [39]. Recently, a comprehensive comparison and clustering of more than two million predicted structures was performed using the Foldseek cluster, and 2.3 million clusters were generated; 53% of them had no structural homologs with experimental structures [40].

As shown in these examples, the structural comparison of AF2 models provides clues for remote homology detection; however, several issues still require clarification. First, the prediction accuracy required for remote homolog detection must be specified. Previous studies on structure comparison of AF2 models used pLDDT (predicted local difference distance test) scores [25] to extract high‐confidence predicted models. Akdel et al. used a pLDDT score > 70 as the threshold [32]. Bordin et al. used several conditions to determine the quality of predicted models, including secondary structure contents and contact densities of residues, in addition to a high pLDDT score [33]. Schaeffer et al. [36] employed the DPAM tool [37] to extract confident structural domains using the predicted aligned error (PAE) [25], residue‐residue distance, hits by HHsuite, and the 3D structural comparison program Dali [19, 20, 41]. However, whether these conditions are sufficient for accurate homolog detection must be clarified. Second, it is unclear whether the predicted 3D structures can aid in recognizing homology not detected by sequence and hidden Markov model (HMM) comparisons. If the query protein has a close homolog in the PDB, it can be classified using BLAST, and experimental or predicted 3D structures are unnecessary. Thornton et al. reported that the pLDDT distribution is heavily skewed toward higher scores for human proteins, where at least a portion of the sequence can be modeled using homology from a structure in the PDB [42, 43]. For proteins with highly confident predictions, 3D structure comparison with the predicted model can work adequately; however, sequence and HMM comparison may be sufficient for detecting homology, as pLDDT scores correlate with the presence of sequence homologs in the PDB.

This study used the ECOD database to evaluate the contributions of AF2‐predicted to homology detection. We chose the ECOD database as the correct standard because it employs manual classification by human experts and provides classifications for relatively recent PDB entries [10]. While the SCOP2 and SCOPe databases also use manual classification, they are not updated frequently [6, 7]. In contrast, the CATH database is updated more frequently; however, since it adopts machine‐learning‐based classification methods in its CATH‐AlphaFlow protocol, its suitability as the correct standard for classification remains uncertain [35].

**FIGURE 1:**
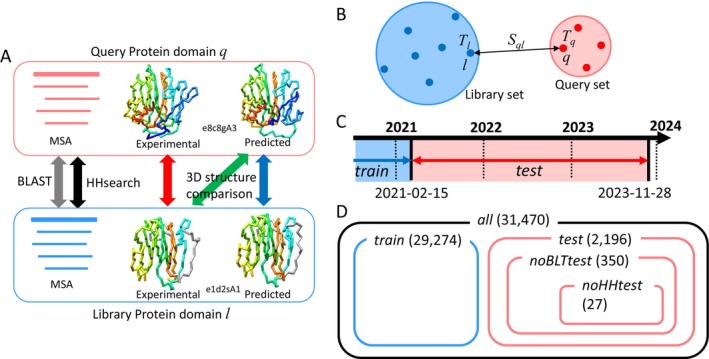
(A) Schematic view of MSA and 3D structure comparison between query and library protein domains. An unclassified new protein is denoted as “query” and a known classified protein as “library.” (B) Calculation of precision and recall between the query (red) and the library (blue) set of protein domains. Domain pairs between the query and the library set were evaluated; those within the query set and the library were not evaluated. (C) The “test” set (blue) and “train” set (red) are defined by the release dates of PDB data. The “test” set is used as the queries, and the “train” set is used as the library. (D) A Venn diagram showing the relationships among the datasets (all, train, test, noBLTtest, noHHtest). The numbers in parentheses indicate the number of domains included in each dataset.

**FIGURE 2:**
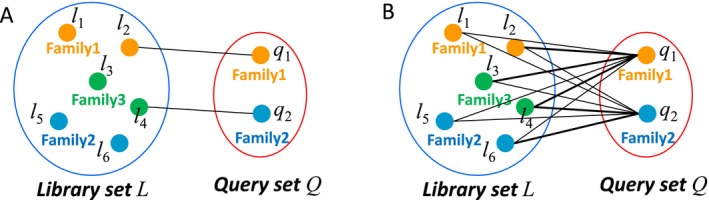
Two statistics to evaluate the performance of homology detection. In this example, the query set *Q* contains two domains and two families, and the library set *L* contains six domains and three families. (A) Statistics of query domains. The performance is evaluated by the ratio of correctly‐assigned query domains. Top‐1 accuracy *A* is affected by only the closest library domain for each query domain; it is not directly affected by other domain pairs. In this case, the family of *q*_1_ is correctly assigned as family 1, and that of *q*_2_ is incorrectly assigned as family 3. Therefore top‐1 accuracy *A* is 1/2 = 0.5. (B) Statistics of query‐library domain pairs. The performance is evaluated by the ratio of correctly‐assigned query‐domain pairs. In this case, there are 12 query‐domain pairs. Among these, four pairs share the same family classification, five pairs (shown in bold lines) have a similarity score greater than a given threshold value *S*. Among the five pairs, only two share the same family. In this case, precision *P*(*S*) = 2/5 = 0.4, recall *R*(*S*) = 2/4 = 0.5, and *F*_1_‐score is 1/(1/0.4 + 1/0.5)/2) = 0.44. As shown in this example, the *F*_*β*_‐score reflects the performance in detecting more remote relations compared to top‐1 accuracy. Details of the definition of *F*_*β*_‐score are described in Section 4.

First, the AF2‐predicted structures corresponding to each domain registered in ECOD were downloaded from AFDB. Then, using both the experimental and predicted 3D structures, we performed 3D structure comparisons with MATRAS [44], Dali [19, 20, 41], and Foldseek [45]; sequence comparisons using BLAST [17], as well as HMM‐HMM comparisons using HHsearch [18] (Figure 1A). The datasets were then classified into query and library sets, and top‐1 accuracy and *F*_*β*_ values were used to evaluate the performance (Figure 1B). We used two different definitions for the query and library sets: (1) a blind test of the predicted model was conducted focusing on the PDB release date (Figure 1C,D), and (2) a dataset classified by the confidence scores of pLDDT was used to investigate the effect of prediction accuracy on homology recognition. To assess the confidence intervals and the statistical significance of these scores, we performed bootstrap samplings. We also investigated how the experimental methods (such as x‐ray, NMR, and EM) that determined the experimental structures affected the performance of homology detections using the corresponding predicted structures.

## 2 Results and Discussion

### 2.1 Overview of Dataset Preparation

In this study, “query” and “library” proteins using “experimental” and “predicted” structures were compared, as shown in Figure 1A. We denoted an unclassified new protein as a “query” and a known classified protein as a “library.” All similarities between the domains in the query and library sets were calculated, and homology detection performances were evaluated with respect to the homology described in the ECOD database as the correct standard. To detect a homologous relationship for a pair of proteins, we considered three types of similarities: (1) 3D structure comparisons (MATRAS [44], DALI [19, 20, 41], and Foldseek [45]), (2) standard sequence comparison (BLAST [17]), and (3) HMM‐HMM comparison (HHsearch [18]). We performed 3D structure comparisons for three different combinations using two available types of 3D domain structures: experimental 3D structures registered in ECOD and predicted 3D structures registered in AFDB. The three different combinations were experimental versus experimental (ECOD vs. ECOD), predicted versus experimental (AFDB vs. ECOD), and predicted versus predicted (AFDB vs. AFDB). Predicted versus experimental comparisons were the primary focus of this study. In contrast, conventional structure classification uses experimental vs. experimental comparisons and was expected to yield the best performance. Considering the blind‐test setting of the query and library set definition, five different datasets of protein domains (“all,” “train,” “test,” “noBLTtest,” and “noHHtest”) were prepared (Table 1; Figure 1D). The “train” dataset comprised PDB structures released before February 15, 2021, while the “test” dataset consisted of PDB structures released after February 15, 2021, respectively (Figure 1C). The “noBLTtest” set was the subset of the test dataset (350 domains) with no homology detected by BLAST with a threshold *E* value of 0.001 against the train dataset. The “noHHtest” set was the subset of the test dataset (27 domains) with no homology detected by BLAST and HHsearch with a threshold *E* value of 0.001 against the train set. The noBLTtest and noHHtest datasets are challenging for detecting homology with BLAST and HHsearch. The relationship among the datasets is shown as a Venn diagram (Figure 1D). Note that, as will be shown later, both BLAST and HHsearch can recognize homologs to some extent in the noBLTtest and noHHtest datasets (Top‐1 accuracy and *F*‐scores were greater than zero), since the *E* value of 0.001 is a conservative threshold. Several combinations for the query and the library sets were evaluated, such as “test‐vs‐train,” “noBLTtest‐vs‐train,” and “noHHtest‐vs‐train”. The “test‐vs‐train” was the comparison between 2196 domains in the “test” set against 29 274 domains in the “train” set (query: “test” set; library: “train” set). These comparisons are realistic for determining the performance of the predicted 3D structures because the predicted 3D structures in the query dataset were generated without knowing the correct 3D structures. The “noBLTtest‐vs‐train” comprised the comparison of 350 domains in the “noBLT test” set against the “train” set (query: “noBLTtest” set; library: “train” set). The “noHHtest‐vs‐train” comprised the comparison of 27 domains in the “noBLT test” set against the “train” set (query: “noHHtest” set, library: “train” set). The latter two comparisons are more realistic for using predicted 3D structures because if a clear homolog is found for the query protein by BLAST or HHsearch against known 3D structures, most structural classification databases classify the query protein into the group of the found homologous structure without using 3D structural information. Note that due to the small number of only 27 domains in the noHHtest dataset, the statistical robustness of the comparison “noHHtest‐vs‐train” will be low. Domain names and related information about the datasets are provided as an online repository (https://doi.org/10.5281/zenodo.14523133).

**TABLE 1:** The datasets of 3D structures of protein domains used in this study.

Data set	Explanation	Ndomains
*all*	The subset of ECOD (20231128) F40 representative 48 933 domains. Details are described in Table S1.	31 470
*train*	The subset of the “all” set that was released before 2021‐02‐15. This dataset should be a training set for AlphaFold2.	29 274
*test*	The subset of set “all” set that was released after 2021‐02‐15. This dataset should be a blind test set for AlphaFold2.	2196
*noBLTtest*	The subset of the “test” set containing proteins without homology detected by BLAST with a threshold *E* value of 0.001 against the PDB proteins publicly released before 2021‐02‐10.	350
*noHHtest*	The subset of the “test” set without homology detected by BLAST and HHsearch with a threshold *E* value of 0.001.	27

*Note:* In this study, we refer to unclassified domains as the “query” and classified domains as the “library.” The choice of which dataset serves as the query and which as the library depends on the evaluation criteria. For example, in “noBLTtest‐vs‐train” (Figure 3), the noBLTtest set serves as the query, while the train set serves as the library.

### 2.2 Two Types of Metrics to Evaluate Homology Detection Methods

We employ two metrics to evaluate homology detection performance: top‐1 accuracy and the *F*_*β*_‐score. Top‐1 accuracy is defined as the ratio of correctly classified query domains, where each query domain is assigned the taxonomy of the library domain with the highest similarity score (Figure 2A). This metric is particularly relevant because most classification tools primarily focus on the top‐1 hit. In contrast, the *F*_*β*_‐score based on the precision‐recall plot considers all query‐library pairs in the dataset, rather than focusing solely on the pair with the highest score, as shown in Figure 2B. Details of the definition of *F*_*β*_‐score are described in the Section 4. Consequently, the *F*_*β*_‐score reflects the performance in detecting more remote homologs compared with top‐1 accuracy. The precision‐recall plot required for *F*_*β*_‐scores used in this study differs from the conventional one because it is calculated considering the similarity only between the query and library datasets (Figure 1B). The plot was further evaluated using three *F*_*β*_‐scores, *F*_1_, *F*_0.5_, and *F*_2_, which are defined as weighted harmonic means of recall and precision. Score *F*_1_ is the harmonic mean with equal weights for recall and precision, whereas score *F*_0.5_ emphasizes precision over recall. Conversely, score *F*_2_ shows more emphasized recall than precision.

### 2.3 Evaluation Using Test Set and Train Sets Classified by Their Release Date

**FIGURE 3:**
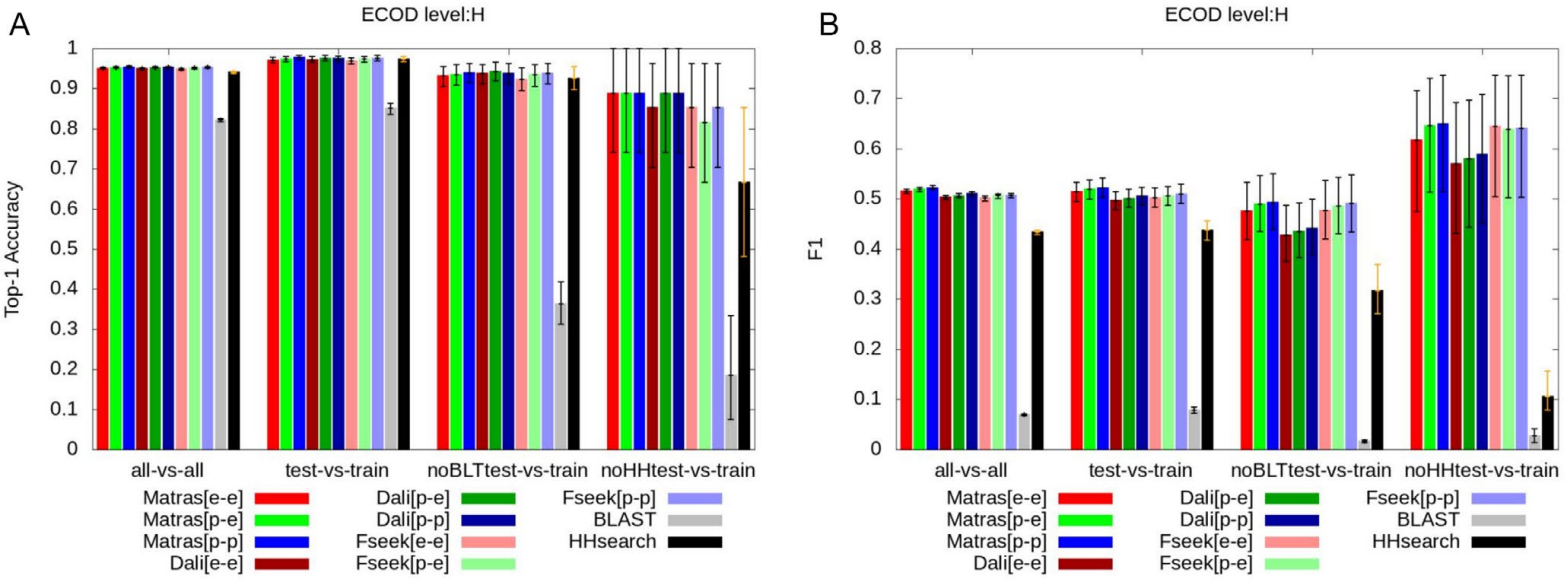
Performances of various sequence and structure comparisons to discriminate between ECOD “H” levels and non‐“H” levels for different combinations of the query and library sets. (A) Top‐1 accuracy of various datasets, methods, and structures. (B) *F*_1_‐scores of various datasets, methods, and structures. “Fseek” stands for the program Foldseek. “[e–e],” “[p–e],” and “[p–p]” indicate comparisons between experimental and experimental structures, those between predicted and experimental structures, and those between predicted and predicted structures, respectively. The error bars in the bar graphs indicate the 95% confidence intervals estimated using the bootstrap method.

Figures 3, S1, and S2 summarize several homology detection comparisons using the ECOD database. Five key points are highlighted here. First, 3D structure comparisons and HHsearch were nearly equal in top‐1 accuracy for all‐vs‐all, test‐vs‐train and noBLT‐vs‐train dataset pairs (Figure 3A). Even the comparison of experimental vs. experimental structures was not significantly superior to HHsearch. In the comparison between noHHtest and train, the Top‐1 accuracy of HHsearch was lower than those of MATRAS structural comparisons (*p* value = 0.028–0.036). This is a natural outcome, considering the definition of the noHHtest set. On the other hand, the *F*_1_‐scores based on domain pair‐based statistics, as shown in Figure 3B, indicate different trends. *F*_1_‐scores for 3D structure comparisons were significantly higher than those for HHsearch across all four dataset pairs. This difference was attributed to the *F*_1_‐scores reflecting more distant relationships between query and library domains (Figure 2B).

The results can be summarized as follows. For most of the newly identified proteins released after February 15, 2021, classification could be performed with high accuracy using only the Top‐1 hit from HHsearch, making 3D structural comparison (even with experimental structures) unnecessary. On the other hand, 3D structure comparison was more effective for recognizing relationships with more distantly related proteins. These remote relationships appeared in the query‐library domain pairs with Top‐*n* hits (*n* > 1). We expect that if a search is conducted using all predicted structures from AlphaFoldDB as a query, there will be many predicted structures in which such distant library homologs appear as the Top‐1 hit.

Second, in 3D structural comparison methods, almost no significant differences in scores were observed between the all‐vs‐all, test‐vs‐train, and noBLTtest‐vs‐train comparisons, both for top‐1 accuracy and *F*_1_‐score. This indicates that AlphaFold2 provides sufficiently accurate predicted structures even for proteins with experimental structures released after February 15, 2021, and even when limited to proteins with no homologs found by BLAST. In contrast, for sequence comparison methods (BLAST and HHsearch), scores for noBLTtest‐vs‐train were slightly but significantly lower than those for test‐vs‐train. This was a natural result, considering the definition of the noBLTtest dataset. It is puzzling that the *F*_1_‐score of noHHtest‐vs‐train is higher than that of test‐vs‐train. This may be due to a family bias caused by the small data size of the noHHtest dataset (27 domains).

Third, the performances of three structural comparison programs—MATRAS, Dali, and Foldseek—were not significantly different using top‐1 accuracy and *F*_1_ score. Fourth, when comparing the precision‐focused *F*_0.5_ score (Figure S1C), there were no large differences between HHsearch and structural comparisons. However, regarding the recall‐focused *F*_2_ score (Figure S1D), the performance of 3D structural comparisons was largely better than that of HHsearch. These observations suggested that structural comparisons can recognize more homologs (higher recall) with lower reliability (lower precision) than HHsearch. In addition, for X‐level recognition, 3D structure comparison outperformed HHsearch in terms of both *F*_0.5_ and *F*_2_ (Figure S2), indicating that 3D structure comparisons can recognize more distant homologs and analogs (X‐level relationships) than HHsearch.

**FIGURE 4:**
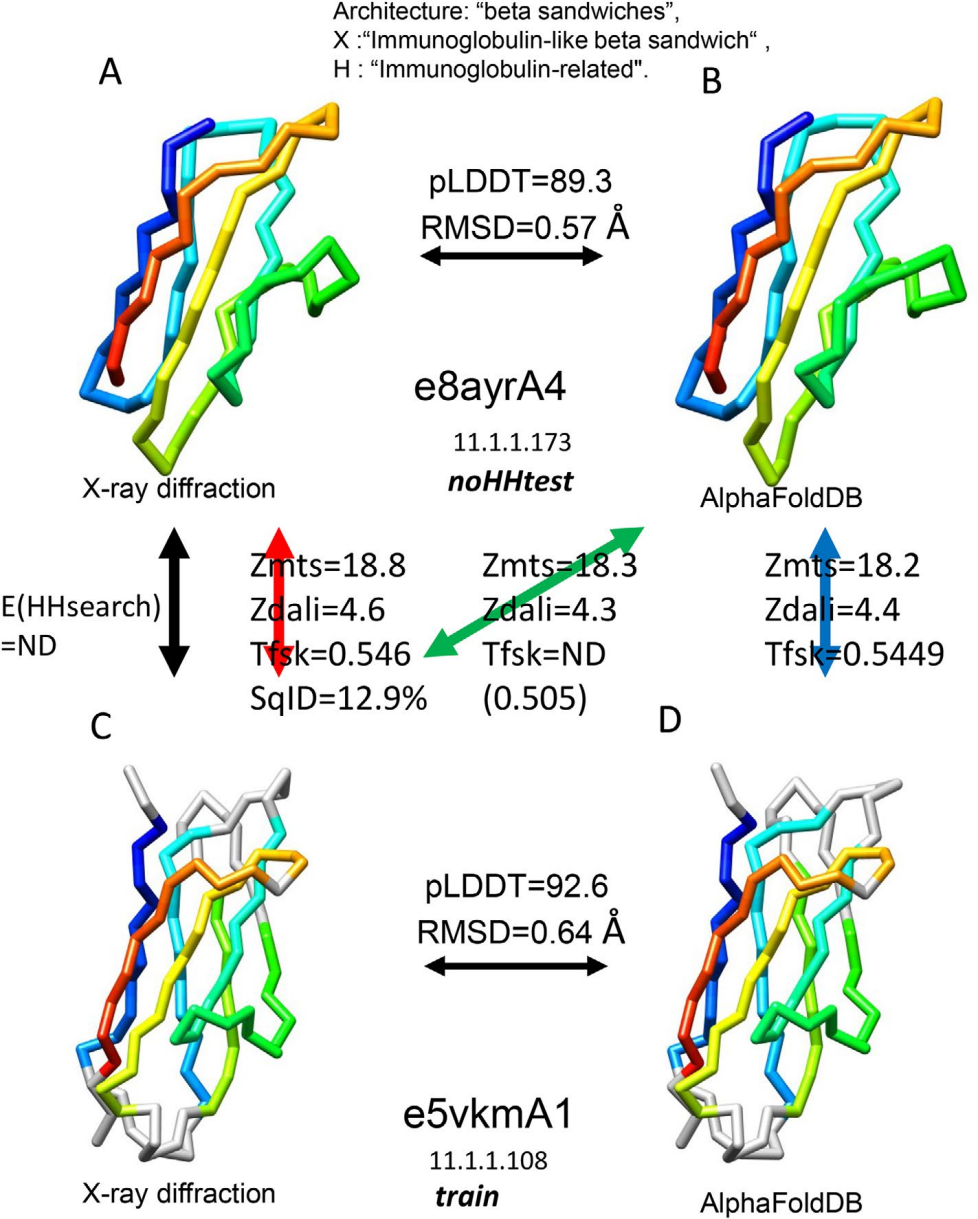
A successful example where a structural comparison of a predicted structure recognized distant homology. In this case, the predicted structure shows performance comparable to experimental structures. HHsearch failed to recognize this homology. (A) An experimental structure and (B) a predicted structure for the query protein domain e8ayrA4. (C) An experimental structure and (D) a predicted structure for the library protein domain e5vkmA1.

Fifth, the performances of structural comparisons among the experimental structures (red bars), between the predicted and experimental structures (green bars), and among the predicted structures (blue bars) showed no significant difference. This suggested that most of the predicted structures in the AFDB, even in the noBLTtest and noHHtest datasets, were sufficiently similar to the corresponding experimental structures. Successful examples are presented in Figure 4 and S9, showing predicted and experimental 3D structures of the query and homologous libraries of proteins. The structural similarity between the predicted query structure and the homologous library experimental structure was higher than the threshold value of the similarity score that maximizes the *F*_0.5_‐score. These high homology detection performances can be explained by their high accuracy of 3D structures. If the predicted 3D structures are similar to the experimental 3D structures, their homology detection performances should be similar. The score thresholds for each method can be downloaded from the online repository (https://doi.org/10.5281/zenodo.14523133). These values will serve as useful guidelines for classifying proteins using structural comparison methods.

### 2.4 The Confidence Measure of Predicted Structures (pLDDT) Affects Homology Detection

**FIGURE 5:**
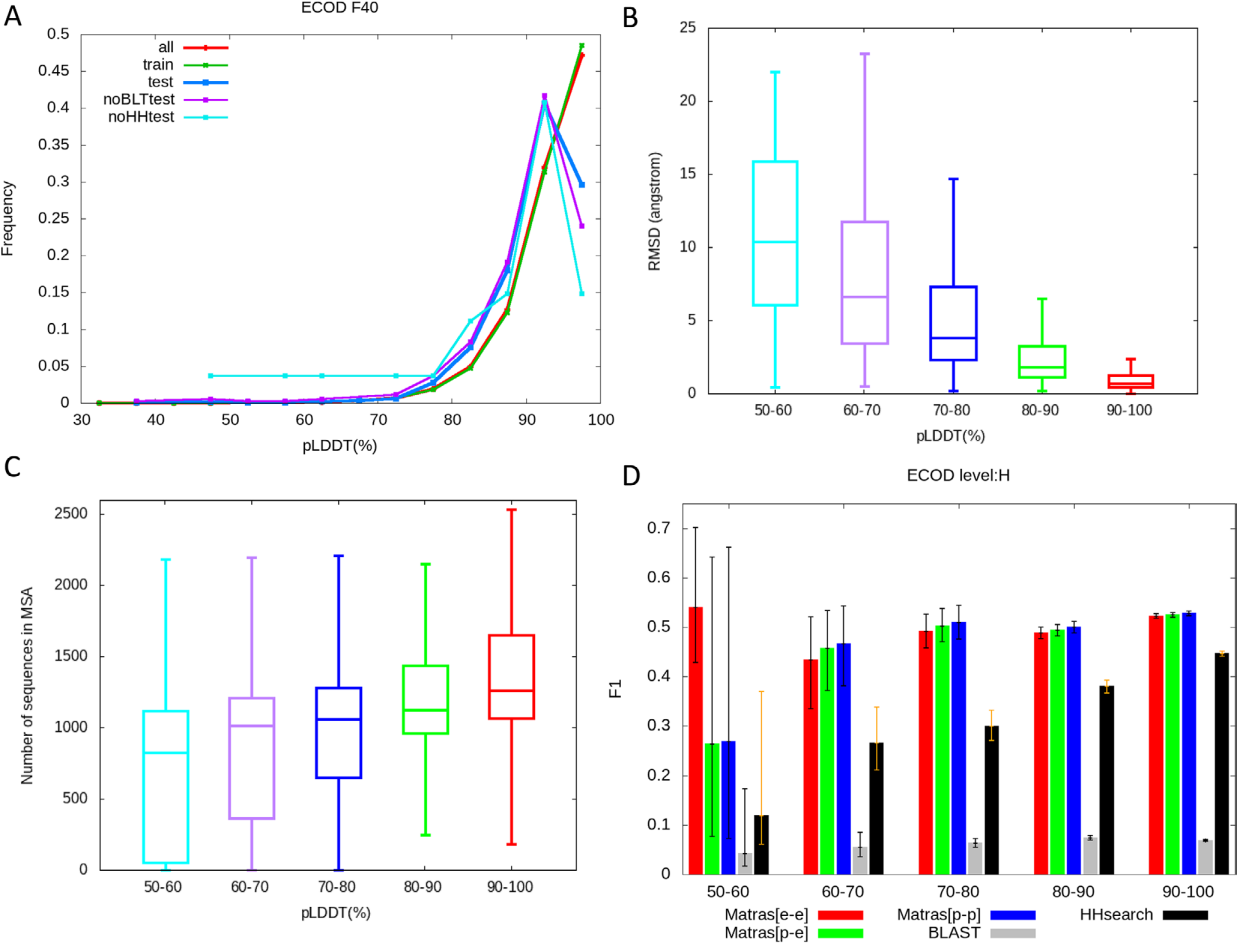
Distribution of pLDDT values for predicted structures. (A) Distribution of pLDDT values for four sets of domains with a bin of width 5%. (B) A box plot of RMSD between predicted and experimental structures for different ranges of pLDDT. (C) A box plot of the number of homologous sequences identified by the program *hhblits* for different ranges of pLDDT. (D) *F*_1_‐scores of MATRAS, BLAST, and HHsearch to discriminate between ECOD “H” levels and non‐“H” levels for five different pLDDT value query sets against the “all” library set. The error bars in the bar graphs indicate the 95% confidence intervals of *F*_1_‐scores estimated using the bootstrap method. Corresponding graphs for Dali and Foldseek are shown in Figure S4.

Next, we investigated whether the pLDDT score, a measure of confidence in the predicted structures, affected homology recognition. Table S2 and Figure 5A show the distributions of pLDDT scores for the five datasets. Figure 5B shows that the pLDDT score was strongly correlated with the root mean square deviation (RMSD), indicating that pLDDT is a reliable measure of prediction accuracy. The pLDDT values in the “all” set and the “train” set were distributed with a very high value, and the bin with pLDDT> 90 was the most common. This is because most domains of these datasets have corresponding experimental structures, and AlphaFold2 used them during training. In contrast, the pLDDT values in the “test,” “noBLTtest,” and “noHHtest” set peaked at slightly lower values (Figure 5A). This is similar to the distribution of PDB structure available human proteins reported by Thornton et al. (2021) [42]. The “noBLTtest” set is thought to correspond with No PDB structure in Thornton et al. (2021); however, it was distributed with higher values of pLDDT (Figure 5A), presumably because the protein structures stored in the ECOD database have few intrinsically disordered regions.

To estimate the impact of pLDDT values on homolog discrimination performance, we classified and studied the “all” dataset by the value of pLDDT. In view of the blind test, we should study the “test” dataset; however, the size of the “test” dataset was too small for stratification statistics, especially in the small pLDDT values, as shown in Table S2. Therefore, we extracted the domains in the “all” set (31 470 domains) into five query subsets using residue‐averaged pLDDT values (“50–60,” “60–70,” “70–80,” “80–90,” and “90–100”) (Figure 1E and Table S2). Figure 5D summarizes the performances of MATRAS, BLAST, and HHsearch using different query sets of pLDDT scores to detect homologies based on the *F*_1_ score. The performances obtained using the other two structural comparisons (Dali and Foldseek) are shown in Figure S4.

Three key points are highlighted in Figures 5D and S4. First, as the pLDDT value decreased from 100 to 60, the corresponding *F*_1_‐score of 3D structure comparisons using predicted structures (“pred‐vs‐exp” or “pred‐vs‐pred”) gradually declined, and as pLDDT values fell below 60, the *F*_1_‐score largely declined (Figure 5D). The decreases of the *F*_1_‐score of MATRAS (pred‐vs‐exp) from pLDDT 90–100 for 80–90 and 60–70 were small but statistically significant (the corresponding *p* values were 0.000 and 0.041). On the other hand, the difference in *F*_1_‐score (pred‐vs‐exp) for pLDDT for 50–60 from 90 to 100 was large, but its *p* value was not sufficiently low (*p* = 0.079); this was due to the small data size of the dataset 50–60 (30 domains). A similar trend was observed for top‐1 accuracy (Figure S3A) and other 3D comparison methods (Dali and Foldseek) (Figure S4).

Secondly, in the range of 50–60, the performance of the predicted structures was largely lower than that of the experimental structures, although its *p* value was not sufficiently low (*p* = 0.080). This is a natural consequence, given the lower predictive accuracy of the predicted structures. Examples illustrating these cases are shown in Figures S10 and S11. In contrast, in the range of 60–100, the performances of exp‐vs‐exp comparisons are not significantly larger than those of pred‐vs‐exp and pred‐vs‐pred. On the contrary, the *F*_1_‐scores were highest for pred‐vs‐pred, followed by pred‐vs‐exp, and lowest for exp‐vs‐exp across the dataset 60–70, 70–80, 80–90, and 90–100. Although these differences were not statistically significant, it was puzzling why the *F*_1_‐scores for exp‐vs‐exp comparisons were not the highest. We will discuss this point later.

Third, the performance of HHsearch largely deteriorated as the pLDDT scores decreased across the range of 100–50. These differences were statistically significant. The fact that HHsearch's performance, which does not rely on any information regarding the 3D structure, correlates with the predicted structure accuracy (pLDDT) appears counterintuitive. The correlation between pLDDT and HHsearch performance may occur because both depend on the number of homologous sequences. The domains with lower pLDDT values had fewer homologous sequences (Figure 5C). Therefore, the pLDDT score is expected to be low, and the HHsearch performance would be poor if there were few homologous sequences for the query protein. For query proteins with low pLDDT values (a few homologous sequences), both 3D comparisons with predicted structures and HMM‐HMM comparisons did not effectively recognize homologs.

### 2.5 The Experimental Method Affects the Properties of Predicted Structures

The experimental structure was expected to be more accurate than the predicted structure. Therefore, we expected that the experimental structure would exhibit better homology recognition. However, Figures 3 and 5D show that the experimental structure (red bars) was not better than the predicted structure (green and blue bars) except in the case of very low pLDDT (50 < pLDDT < 60) versus all. On the contrary, the predicted structure showed better performance, particularly when the pLDDT value was between 60 and 70, although this superiority was not sufficiently significant (Figures 5D and S3A; *p* values for pred‐vs‐pred is better than exp‐vs‐exp were and 0.043 for top‐1 accuracy and 0.309 for *F*_1_‐score). This trend was observed not only in MATRAS but also in Dali and Foldseek (Figure S4).

**FIGURE 6:**
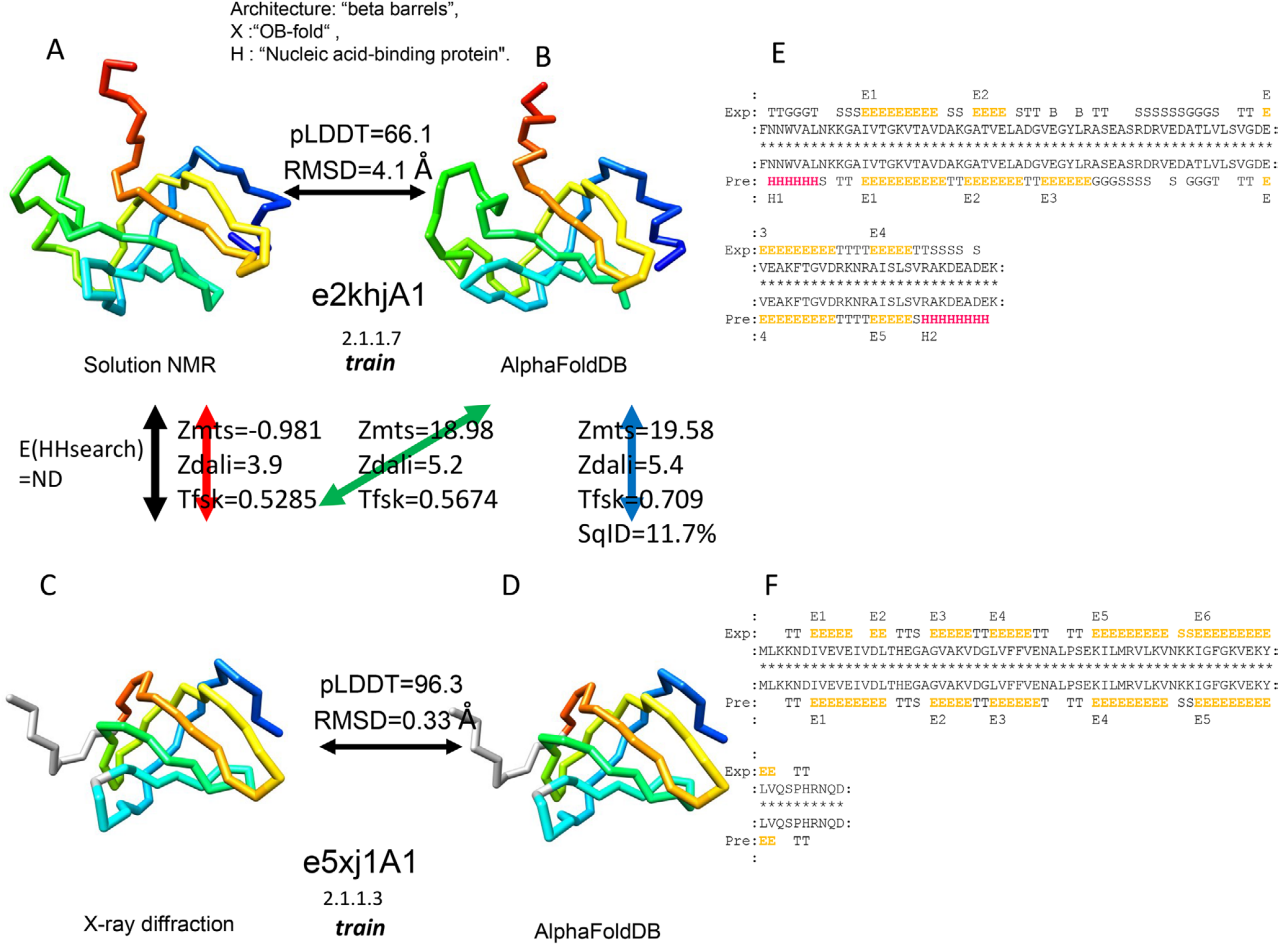
An example where structural comparison using predicted structures showed a better performance for homology recognition than the experimental structure. Solution NMR determined the query experimental structure. The predicted structure of the query contains more residues in α helices and β strands compared to the experimental structure. (A) An experimental structure and (B) a predicted structure for the query protein domain e2khjA1. (C) An experimental structure and (D) a predicted structure for the library protein domain e5xj1A1. (E) An alignment between the experimental and predicted structure for the query domain. (F) An alignment between the experimental and predicted structure for the library domain.

**FIGURE 7:**
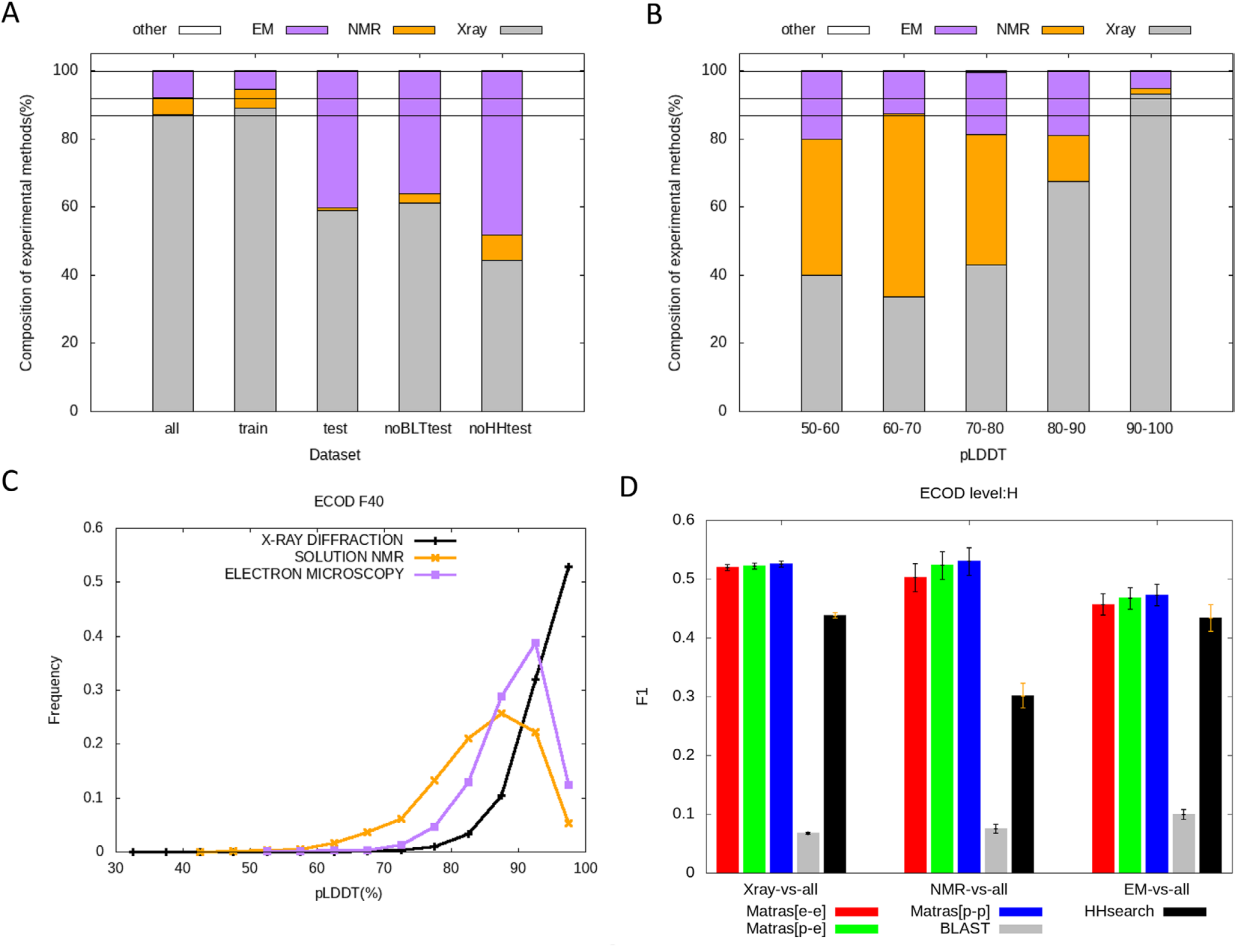
Distribution of three major experimental methods for experimental structures. (A) Compositions of experimental methods for the five datasets. (B) Compositions of experimental methods for different ranges of pLDDT of corresponding predicted structures. (C) Distributions of the pLDDT values of predicted structures for three different experimental methods of corresponding experimental structures. (D) *F*_1_‐scores of MATRAS, BLAST, and HHsearch to discriminate between ECOD “H” levels and non‐'H' levels for the three different sets of experimental methods versus the “all” dataset. The error bars in the bar graphs indicate the 95% confidence intervals of *F*_1_‐scores estimated using the bootstrap method. Corresponding graphs for Dali and Foldseek are shown in Figure S5.

After observing several cases where the experimental structures were inferior, we noticed that such cases were often observed in structures determined by nuclear magnetic resonance (NMR) or electron microscopy (EM). Examples are shown in Figures 6, S12, and S13. We investigated how the method used to determine the experimental structure affected homology recognition by extracting the three subsets using experimental methods. X‐ray (27 366 domains), NMR (1583 domains), and EM (2490 domains) datasets were extracted from the “all” set, corresponding to the experimental methods of x‐ray diffraction, solution NMR, and electron microscopy. Figure 7A summarizes the compositions of the three experimental methods for the five datasets. Compared to the “all” and “train” datasets, the “test,” “noBLTtest,” and “noHHtest” datasets had fewer “Xray” and more “EM” entries. This is a reasonable result, considering the recent advances in electron microscopy. Figure 7B,C, show the interesting relationship between pLDDT and experimental methods. When the pLDDT scores were very high (90–100), almost all experimental structures were determined by x‐ray crystallography. However, as the pLDDT score decreased, the proportion of structures determined by NMR increased by more than tenfold, while that determined by electron microscopy also increased several times.

Using each of the three sets (“x‐ray,” “NMR,” and “EM”) as queries and the “all” set as a library, the *F*_1_‐score was calculated (Figure 6D). In “Xray‐vs‐all,” the *F*_1_‐scores for the three combinations (exp‐vs‐exp, pred‐vs‐exp, and pred‐vs‐pred) were almost the same. On the other hand, in “NMR‐vs‐all”, the *F*_1_‐score for exp‐vs‐exp (0.503) was much lower than that for pred‐vs‐pred (0.530), although its *p* value = 0.060 was marginally significant. This trend was observed not only in MATRAS but also in Dali and Foldseek (Figure S5).

**FIGURE 8:**
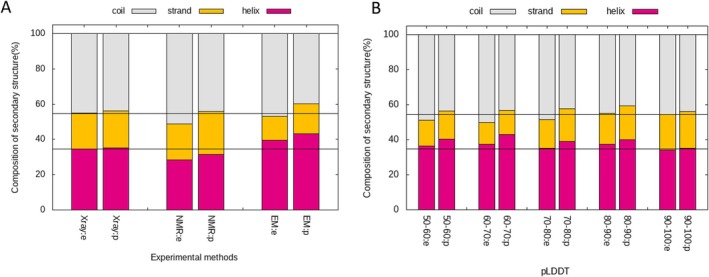
Compositions of experimental and predicted secondary structures for various datasets. Compositions of experimental structures are denoted as “:e,” whereas those of predicted structures are denoted as “:p.” (A) Compositions of secondary structures for the three different sets. (B) Compositions of secondary structures for different ranges of pLDDT.

Next, we examined the secondary structure compositions for experimental and predicted structures (Figure 8A). The compositions of predicted and experimental structures, as determined by x‐ray crystallography, are similar. However, for proteins determined by NMR and EM, the AF2‐predicted models had more regular secondary structures (helix or strand) than the experimental structures; the compositions of the AF2 predictions for NMR‐determined experimental structures were similar to those of the X‐ray experimental structures (Figure 8A). In regions with low pLDDT scores, the predictions tended to have more regular secondary structures than the experiments (Figure 8B).

Figure S6 summarizes the distributions of the number of amino acids and sequences in the multiple sequence alignment (MSA) for each experimental method and the range of pLDDT. Domains determined by NMR or with smaller pLDDT values tended to have fewer amino acids and sequences in the MSA. Figure S7 summarizes the distributions of RMSD between experimental and predicted structures and the ratio of residues predicted as “disordered” from the amino acid sequence by the program DISOPRED 3.1 [46]. Domains determined by NMR or EM and with smaller pLDDT values tended to have larger RMSD and more residues predicted as a disorder. Figure S6 suggests that because proteins determined by NMR or EM have more unstable disorder‐like regions, they are difficult to determine correctly experimentally, and the deviation between experimental and predicted structures is large.

Based on the findings presented in Figures 7, 8, S6, and S7, we can infer why the predicted structures were better at recognizing homologous structures than the experimental structures determined by NMR. Suppose for NMR‐determined proteins, AlphaFold2 tends to predict structures closer to those determined by x‐ray; in that case, comparing predicted and experimental structures would be better than comparing experimental and experimental structures because most library domain structures are provided by x‐ray. In other words, the structures predicted by AlphaFold2 exhibited a certain level of commonality, tending to average and standardize structural differences across experimental methods.

### 2.6 Establishing a New Homologous Group for a Predicted Structure Without Classification Into a Known Group

Proteins with predicted structures that have no homology to any known three‐dimensional structure groups were reported by several studies [33, 40]. In such cases, it is necessary to establish a new homologous group (level “X” in ECOD database) when classifying these proteins. If the top‐1 hit structure in the library does not have a sufficiently high similarity score, the query structure should be classified into a new group specifically for this query structure. Defining a sufficiently high score is a critical issue in establishing a new group. In this study, we provide multiple threshold values based on different criteria, which can be downloaded from the online repository (https://doi.org/10.5281/zenodo.14523133). We provided threshold values for three structure pair types (exp‐vs‐exp, pred‐vs‐exp, and pred‐vs‐pred), four dataset comparisons (all‐vs‐all, test‐vs‐train, noBLTtest‐vs‐train, and noHHtest‐vs‐train), and five score definitions (precision = 0.99, precision = 0.90, maximum F0.5 score, maximum F1 score, and maximum F2 score). These values serve as useful guidelines for classifying proteins using structural comparison methods.

To evaluate the strategy for establishing a new group, we examined the ECOD domains in our test set released between February 15, 2021 and November 28, 2023 to identify domains assigned to a newly established “X” group. We searched for domains with no homologs in the PDB as of February 02, 2021 using BLAST and HHsearch. Unfortunately, only one domain, e7dc3A1, met these criteria among the 27 domains in the noHHtest dataset. The experimental and predicted structures of this domain are shown in Figure S14. It consists of three *β*‐strands and one long *α*‐helix. The predicted structure (Figure S14C) closely matched the experimental structure (Figure S14B; RMSD = 2.0 Å). This domain e7dc3A1 (release date: August 25, 2021) had no homologs detected by BLAST or HHsearch in the PDB as of February 15, 2021 and was classified as 3240.1.1.2 in the ECOD database. This group was newly established for this domain. The three programs—MATRAS, Dali, and Foldseek—identified three similar structures as the top‐1 hits for the predicted structure; however, their similarity scores were below the threshold for a precision of 0.9 (Figure S14D,E,F). The search results using the experimental structure were similar. Although this is only a single case, it is a valuable example of establishing a new group for novel proteins.

## 3 Conclusions

This study comprehensively explored the sequence, HMM, and 3D structure comparisons among the domain structures registered in the ECOD database using experimental and predicted structures. The structures were classified into test and training sets based on whether the release date of the structure was before or after February 15, 2021. 3D structure comparisons and HHsearch were nearly equal in top‐1 accuracy for test‐vs‐train and noBLT‐vs‐train dataset pairs, whereas 3D structure comparisons performed better in terms of *F*_1_‐score (Figure 3). It suggests 3D structure comparisons are more effective than HHsearch in finding more remote homologs. In 3D structure comparisons, no significant differences were observed between test‐vs‐train and noBLT‐test‐vs‐train comparisons or between experimental and predicted structures comparisons. This is because the prediction accuracy of AlphaFold2 is extremely high, even for structures without close homologs in the train set. The performance of HHsearch declined for query proteins with lower pLDDT values, likely due to the fact that the number of sequences in multiple alignments affected both pLDDT and HHsearch. Furthermore, the type of the experimental methods used to determine the experimental structure was associated with the properties of the corresponding predicted structure. In particular, the predicted structures of proteins determined by NMR had lower pLDDT values and contained more helices and strands compared to their experimental structures. That is why the predicted structures, whose experimental structures were determined by NMR, showed better homology detection performances than the corresponding experimental structures.

In summary, recognizing distantly related homologs by 3D comparison of predicted versus experimental structures with sufficiently high pLDDT showed good results, and a comparison of predicted versus predicted structures worked similarly well. These results provide evidence for the effectiveness of recent studies on family classification using predicted structures [32, 33], as well as comparisons among predicted structures [40]. In other words, if a newly solved experimental structure or a structure predicted by AlphaFold2 with sufficient pLDDT values for a new sequence is available, we should perform a structural search against not only the PDB but also AlphaFoldDB to find its homologs. The performances against experimental structures and those against predicted structures are not so much different. Of course, if similar structures are found within the PDB, richer information, such as 3D details about interacting molecules, can be acquired. However, AlphaFoldDB contains far more structures, which increases the likelihood of finding similar structures. The score thresholds for each method can be downloaded from the online repository (https://doi.org/10.5281/zenodo.14523133). These values will serve as useful guidelines for classifying proteins using structural comparison methods.

## 4 Materials and Methods

### 4.1 3D Models of ECOD and AFDB

The number of domains used in this study is summarized in Tables 1 and S1. We used the F40 representative domains of the ECOD database version 20 231 128 (develop290). First, the domain text file “ecod_develop290.F40.domains.txt” and the PDB files “ecod.develop290.F40.pdb.tar.gz” were downloaded on April 05, 2024, which contained 43 434 domains (“original” set in Table S1). After extracting domains with not less than 40 residues, not less than four secondary structure elements, and not in obsolete PDB entries, 39 813 domains remained (“regular” set in Table S1). We extracted the UniProt accession number (AC) [47] from the struct_ref.pdbx_db_accession of an mmCIF file for the corresponding PDB ID [48] for each domain. Using UniProt ACs, we downloaded the AF2‐predicted model from the AFDB (https://alphafold.ebi.ac.uk/files/AlphaFoldDB_PDB/AF‐[UniProtAC]‐F1‐model_v4.pdb). Because the AF2 model represents the entire protein, its parts corresponding to ECOD domain PDB files were extracted to create a domain model file. Among the 39 813 domains, 31 470 domains had corresponding AF2 models in AFDB to obtain the final representative list of 31 470 domains (“all” set in Table 1). The conditions for the correspondence were as follows: sequence identity is not less than 95%, the ratio of aligned residues (number of aligned residues divided by the length of ECOD domains) is not less than 95%, and the number of corresponding residues is not less than 40. DSSP and BSSP files were generated for experimental and predicted domain structures in the “all” set.

The current AlphaFold2 was trained on the PDB released before April 30, 2018, and used the templates released before February 15, 2021. Therefore, to test the ability of remote homolog detection for proteins of unknown structures, we classified domains in the “all” set into the “train” set (released before February 15, 2021; 31 470 domains) and the “test” set (released after February 15, 2021; 2196 domains) (Table 1 and Figure 1D).

With the help of the HOMCOS server [49], a non‐redundant set (252 283 sequences) and a 40% representative set (44 451 sequences) of PDB were prepared for protein structures released on February 10, 2021. Subsequently, we performed BLAST searches of the “test” set (2196 domains) against the non‐redundant set of PDB (252 283 sequences). Of the 2196 domains, 350 domains did not have BLAST homologs in the PDB as of February 10, 2021, with an *E* value of less than 0.001. These 350 domains were called the “noBLTtest” set (Table 1). Similarly, HHsearch was performed on multiple sequence alignments (MSAs) in the test set (2196 domains) against MSAs in the 40% representative chain set (44 451 sequences) of the PDB as of February 10, 2021. Consequently, only 27 domains did not have BLAST homologs and HHsearch‐homologs with an *E* value of < 0.001 (Table S1). The dataset of these 27 domains can be a good query protein for the blind test. However, due to the very small size of this dataset, obtaining statistically significant results is challenging. Domain names and related information about the datasets are provided as an online repository (https://doi.org/10.5281/zenodo.14523133).

### 4.2 HMM–HMM Comparison Search

We used HH‐suite version 3.3.0 for the HMM–HMM comparison search [17]. The HMM library file UniRef30_2020_06 was downloaded from UniClust (https://uniclust.mmseqs.com/) [50]. For the sequences in the ECOD representative “all” list (31 470 domains) and 40% representative set of PDB on February 10, 2021 (44 451 proteins), HMMs were searched through UniRef30_2020_06 using the program *hhblits* to create MSAs in A3M format [51]. Subsequently, the HMM–HMM searches were performed for each generated MSA for an ECOD domain against the ECOD MSA library using the program *hhsearch*. To evaluate similarity, we employed ‐ log_10_ (*E* value). HMM–HMM searches were also performed for the MSA library of the PDB on February 10, 2021.

### 4.3 Score of 3D Structure Comparison Program MATRAS

We used the 3D structure comparison program MATRAS developed by our group [44]. For structure comparison, the DSSP program (version 2.3.0) [52] was applied to the PDB files for the protein domains to assign secondary structures and calculate the accessible surface area. Finally, the BSSP files (DSSP files with *C*_*β*_ atom coordinates) were generated.

MATRAS primarily uses the log‐odds score *S*_*dis*_(*a*,*b*) for the distance *D*_*ij*_ between *C*_*β*_ atoms to evaluate the structural similarity between proteins *a* and *b* as follows: [44]

(1) $$S_{\mathrm{dis}}\left(a,b\right)=\sum_{i}^{N}\sum_{j\left(>i\right)}^{N}\log\frac{P\left(D_{\mathrm{ij}}^{\left(a\right)}\to D_{\mathrm{ij}}^{\left(b\right)}\right)}{P\left(D_{\mathrm{ij}}^{\left(b\right)}\right)}$$

where *D*_*ij*_^(*a*)^ is the C_β_ distance between the *i*‐th and *j*‐th residues for protein *a*, and *D*_*ij*_^(*b*)^ is that for protein *b*. Recently, the *Z*‐score has been introduced to normalize the log‐odds score *S*_*dis*_(*a*,*b*) by the length of the comparing proteins:

(2) $$Z_{\mathrm{dis}}\left(a,b\right)=\frac{S_{\mathrm{dis}}\left(a,b\right)-m\left(\sqrt{N_{a}N_{b}}\right)}{\sigma \left(\sqrt{N_{a}N_{b}}\right)}$$

where *N*_*a*_ and *N*_*b*_ are the lengths of the proteins *a* and *b*, respectively. The mean *m* and standard deviation *σ* of the score *S*_*dis*_ were estimated by linear regression with the geometric mean of *N*_*a*_ and *N*_*b*_, inspired by the Dali *Z*‐score [20]. Linear regressions using the parameters *A*_*m*_, *B*_*m*_, *A*_*σ*_, and *B*_*σ*_ are defined as follows:

(3) $$m\left(\sqrt{N_{a}N_{b}}\right)=A_{m}\sqrt{N_{a}N_{b}}+B_{m}$$

(4) $$\sigma \left(\sqrt{N_{a}N_{b}}\right)=A_{\sigma }\sqrt{N_{a}N_{b}}+B_{\sigma }$$

Using the scores of the non‐homologous structure pairs, the regressed parameters were estimated to be *A*_*m*_ = 16.170350, *B*_*m*_ = −47.164564, *A*_*σ*_ = 23.503942, and *B*_*σ*_ = −762.928232. The regression was performed using the 40% representative 14 225 domains of SCOPe 2.08 [7], satisfying Naa ≥ 40, Nsse ≥ 4. Scores of all‐vs‐all comparisons of the domains with different “fold” were used for the regression, ignoring cross‐fold pairs within {c.2‐c.5, c.27, c.28, c.30, c.31} and {b.66‐b.70} [45]. We also examined the *R*_*dis*_ score, employed previously [53], defined as *R*_*dis*_ = 2*S*_*dis*_(*a*,*b*)/[*S*_*dis*_(*a*,*a*) + *S*_*dis*_(*b*,*b*)]. The performance of *Z*_*dis*_ was significantly better than *R*_*dis*_, as shown in Figure S8.

In this study, we only handled proteins satisfying Naa ≥ 40. However, when handling proteins with fewer than 40 residues, the following equation must be used; otherwise, there is a risk that the standard deviation may become negative.

(5) $$\sigma \left(\sqrt{N_{a}N_{b}}\right)=A_{\sigma }\max\left[\sqrt{N_{a}N_{b}},40\right]+B_{\sigma }$$

### 4.4 3D Structure Comparison Program Dali

The Dali program is a well‐established structural comparison program with a history of usage [19], and it is also employed for the classification of the ECOD database [10]. The source code for “DaliLite.v5” was used for the calculation [41]. The *Z*‐score was employed as the similarity score for Dali [20], which is similar to Equations (2), (3), and (4), whereas Dali regresses the mean *m* up to the third order of the geometric mean of *N*_*a*_ and *N*_*b*_, and the standard deviation *σ* is approximated as 0.5 *m*.

### 4.5 3D Structure Comparison Program Foldseek

Foldseek is a recently developed structural comparison program utilizing a sequence search technique with structural alphabets to achieve sensitivity and high‐speed computation [45]. We employed the most sensitive TM align mode (alignment‐type 1) among the three options (0:3Di, 1:TMalign, and 2:3Di + AA). The average TM‐score = (qTMscore + tTMscore)/2, provided by the “evalue” column, was employed as the similarity score for Foldseek.

### 4.6 Top‐1 Accuracy

Top‐1 accuracy is a straightforward metric to evaluate a classification method, as illustrated in Figure 2A. Top‐1 accuracy *A* is defined as the ratio of correctly classified query domains, where a query domain is classified as the taxonomy of the library domain with the highest similarity score:

(6) $$A\left(Q,L\right)=\frac{N\left(q\in Q;T_{\hat{l}\left(q\right)}=T_{q}\right)}{N\left(q\in Q\right)}$$

where *Q* is the set of query domains, *L* is the set of library domains, and *T*_*q*_ is the ECOD taxonomy of query domain *q*. *N*(*c*) is the number of domain pairs satisfying condition *c*. The library domain $\hat{l}\left(q\right)$ with the largest score *S*(*q*, *l*) for the query domain *q* is defined as follows:

(7) $$\hat{l}\left(q\right)=\underset{l\in L,l\neq q}{\text{argmax}}\left[S\left(q,l\right)\right]$$

Note that when assigning the library domain $\hat{l}\left(q\right)$, an identical library protein (*l* = *q*) is excluded.

### 4.7 Precision–Recall Plot and *F*_*β*_‐Score

To draw a precision‐recall plot for evaluating the performance of similarity scores, the similarity scores for all pairs of domains within the dataset should be calculated. However, in this study, the target domain to be classified (query) and the reference domain (library) have different characteristics. The query domain to be classified may only be available as a predicted structure, whereas the reference library domain is available as both experimental and predicted structures. Therefore, two separate datasets, the query set *Q* and the library set *L*, were prepared. Only the similarity scores between the two sets, *Q* and *L*, were calculated, and the scores within each set were not considered, as illustrated in Figure 2B.

Recall *R*(*S*) and precision *P*(*S*) are defined for the threshold score *S* as follows:

(8) $$R\left(S\right)=\frac{N\left(S_{\mathrm{ql}}>S\cap T_{q}=T_{l}\right)}{N\left(T_{q}=T_{l}\right)}$$

(9) $$P\left(S\right)=\frac{N\left(S_{\mathrm{ql}}>S\cap T_{q}=T_{l}\right)}{N\left(S_{\mathrm{ql}}>S\right)}$$

where *S*_*ql*_ is the score between the query domain *q* (q∈Q) and the library domain *l* (l∈L). *T*_*q*_ is the ECOD taxonomy of the query domain *q*, and *T*_*l*_ is that of the library domain *l*. *N*(*c*) is the number of domain pairs satisfying condition *c*. Note that when calculating the number *N*(*c*), pairs of identical proteins (*q* = *l*) are excluded. If we plot the recall *R*(*S*) and precision *P*(*S*) values for all possible scores *S*, we can obtain a precision‐recall curve, as shown in Figures S1A and S2A.

Precision and recall have a trade‐off relationship in which an increase in one leads to a decrease in the other. The *F*‐score *F*_*β*_ is commonly used to evaluate the balance between these two quantities using a weighted harmonic mean:

(10) $$F_{\beta }=\max_{S}{\left[\frac{1}{1+\beta^{2}}\left(\frac{1}{P\left(S\right)}+\frac{\beta^{2}}{R\left(S\right)}\right)\right]}^{-1}$$

where a parameter *β* is a positive constant indicating the weight of recall [54]. In the *F*_1_ score with *β* = 1, the precision and recall have equal weights. In this study, the *F*_1_ score (*β* = 1) was mainly used; however, the *F*_0.5_ score (*β* = 0.5) with a high precision weight, and the *F*_2_ score (*β* = 2) with a high recall weight were also employed. Threshold scores, recalls, and precisions corresponding to precision = 0.99, 0.9, or maximizing *F*_0.5_, *F*_1_, and *F*_2_ are provided as an online repository.

### 4.8 Estimation of Confidence Intervals and *p* Values Using the Bootstrap Method

Due to the lack of a suitable parametric model for statistical testing of the top‐1 accuracy and the *F*_*β*_‐score, we used the bootstrap method to calculate the confidence intervals and *p* value [55]. From the query set *Q*, the *b*‐th bootstrap sample for the query set Q*^b^ = {*q*_1_*^b^, *q*_2_*^b^, …, *q*_|*Q*|_*^b^} was obtained by randomly sampling domains |*Q*| times, with replacement. The bootstrap method begins by generating the independent *B* random bootstrap query samples Q* = {Q*^1^, Q*^2^, …, Q*^B^}. For example, with *Q* = {*q*_1_, *q*_2_, *q*_3_, *q*_4_, *q*_5_} and *B* = 3, we might obtain samples Q*^1^ = {*q*_1_, *q*_3_, *q*_1_, *q*_4_, *q*_3_}, Q*^2^ = {*q*_1_, *q*_1_, *q*_4_, *q*_5_, *q*_5_}, and Q*^3^ = {*q*_3_, *q*_3_, *q*_1_, *q*_2_, *q*_2_}. We employed *B* = 1000 in this study. We perform bootstrap sampling on the query set *Q*, but for the library set *L*, we use the original dataset without applying bootstrap sampling. This is because the library set is considered to have a sufficient amount of data compared to the query set. Additionally, applying bootstrap sampling to the library set introduces a problem where the bootstrap average of the top‐1 accuracy *A*(*Q*, L*^b^) almost always becomes lower than the original value *A*(*Q*, *L*) due to the reduced diversity of the bootstrap sample library set L*^b^ compared with the original set *L*.

The confidence intervals of the accuracy and the *F*_*β*_‐score are calculated using the percentile method. The confidence interval of the top‐1 accuracy *A* is determined by *A*_*lower*_(Q*, *L*) and *A*_*upper*_(Q*, *L*), as follows:

(11) $$p\left(A_{\text{lower}}\left(Q^{*},L\right)<A\left(Q^{*},L\right)<A_{\text{upper}}\left(Q^{*},L\right)\right)=1-\alpha$$

where *A*(Q*, *L*) is the accuracy estimated from the set Q* and *L*, and *A*_*lower*_(Q*, *L*) and *A*_*upper*_(Q*, *L*) are lower and upper confidence limits. The parameter *α* is a small positive number. In this study, we employed *α* = 0.05, which corresponds to a 95% confidence interval. Using the *B* random bootstrap query samples Q*^1^, Q*^2^, …, Q*^B^, the corresponding accuracies *A*(Q*^1^, *L*), *A*(Q*^2^, *L*), …, *A*(Q*^B^, *L*) are calculated. These accuracies are sorted in increasing order and stored in the array *A*(Q*,*L*) [1], *A*(Q*,*L*) [2], …, *A*(Q*,*L*)[*B*]. The lower and upper confidence limits *A*_*lower*_ and *A*_*upper*_ are estimated as the following percentiles:

$$A_{\text{lower}}\left(Q^{*},L\right)=A\left(Q^{*},L\right)\left[r_{\text{lower}}\right],$$

(12) $$A_{\text{upper}}\left(Q^{*},L\right)=A\left(Q^{*},L\right)\left[r_{\text{upper}}\right]$$

where *r*_lower_ and *r*_upper_ are the ranks of the lower limit and upper bound respectively, and defined as follows:

(13) $$r_{\text{lower}}=\left\lceil \frac{\mathrm{B\alpha}}{2}\right\rceil ,r_{\text{upper}}=\left\lceil B\left(1-\frac{\alpha }{2}\right)\right\rceil$$

The confidence intervals are useful for indicating the statistical significance of the difference between the two metrics by a simple bar graph. When we estimate the significance of the difference between two metrics *A* ^(*x*)^ of a condition *x* and *A* ^(*y*)^ of a condition *y*, if the two intervals (*A*_lower_^(*x*)^, *A*_upper_^(*x*)^) and (*A*_lower_^(*y*)^, *A*_upper_^(*y*)^) do not overlap, the *p* value for *A* ^(*x*)^ < *A* ^(*y*)^ is always less than *α*. However, note that even if the two intervals overlap, the *p* value for *A* ^(*x*)^ < *A* ^(*y*)^ can still be less than *α* in some cases. Similarly, the lower and upper confidence limits *F*_*β*_,_upper_(*Q*,*L*) and *F*_*β*_,_lower_(*Q*,*L*) are calculated using the Equations ((11), (12), (13)–(11), (12), (13)).

Using the bootstrap samples, we can directly calculate the *p* value *p*(*A* ^(*x*)^ > *A* ^(*y*)^) even if the two confidence intervals do overlap. The *p* value *p*(*A* ^(*x*)^ > *A* ^(*y*)^) for the accuracy *A* of a condition *x* (a similarity score *S*_*x*_ and a dataset *D*_*x*_) being higher than that of a condition *y* (a similarity score *S*_*y*_ and a dataset *D*_*y*_) is calculated as follows:

(14) $$p\left(A^{\left(x\right)}>A^{\left(y\right)}\right)=1-\frac{1}{B}\sum_{b}^{B}I\left(A^{\left(x\right)}\left(Q^{\left(x\right)*b},L^{\left(x\right)}\right)>A^{\left(y\right)}\left(Q^{\left(y\right)*b},L^{\left(y\right)}\right)\right)$$

where *A* ^(*x*)^(*Q* ^(*x*)^*^b^, *L* ^(*x*)^) and *A* ^(*y*)^(*Q* ^(*y*)^*^b^, *L* ^(*y*)^) are accuracies for the *b*‐th bootstrap sample using the target condition *x* and the reference condition *y*, respectively. The indicator function *I*(*X* > *Y*) returns 1 if *X* > *Y*, and returns 0 if *X* ≤ *Y*. Similarly, the value *p*(*F*_*β*_^(*x*)^ > *F*_*β*_^(*y*)^) of the *F*_*β*_‐score are calculated using the Equation (14).

## Author Contributions

**Takeshi Kawabata:** conceptualization, methodology, investigation, writing – original draft, software, funding acquisition. **Kengo Kinoshita:** funding acquisition, supervision, writing – review and editing.

## Conflicts of Interest

The authors declare no conflicts of interest.

## Peer Review

The peer review history for this article is available at https://www.webofscience.com/api/gateway/wos/peer‐review/10.1002/prot.26828.

## Supporting information


Data S1.

Data S2.

## Data Availability

Supplementary Excel files for datasets (SuppExcel1_domain_list.xlsx) and threshold scores (SuppExcel2_score_thresholds.xlsx) are accessible from an online repository (https://doi.org/10.5281/zenodo.14523133). The 3D structures of the representative domains used in this study have been submitted to the Biological Structure Model Archive (BSM‐Arc) under BSM‐ID BSM00057 (https://bsma.pdbj.org/entry/57) [57].
