# Supplementary material for: Assessing Structural Classification Using AlphaFold2 Models Through ECOD‐Based Comparative Analysis
Source: Proteins. 2025 Apr 19;93(9):1571–85. doi: 10.1002/prot.26828 (PMC12314581; doi:10.1002/prot.26828)
Supplement: Supplementary file 1 — Data S1. [file PROT-93-1571-s001.pdf]

Supplementary Information

Assessing Structural Classification Using AlphaFold2 Models through ECOD-based Comparative Analysis

AUTHOR NAME: Takeshi Kawabata[1], Kengo Kinoshita [1]

1: Graduate School of Information Sciences, Tohoku University

LAST UPDATE: 2024/12/15

Table S1. The datasets of 3D structures of protein domains.

Data set	Explanation	Ndomains
original	ECOD (2023/11/28) F40 representatives	43,434
regular	The subset of the "original" set satisfying Naa ≥ 40, Nsse ≥ 4. The obsoleted entries are removed.	39,813
all	The subset of the "regular" set for which their predicted structures are also registered in AlphaFoldDB. Sequence identity is not less than 95 %, the ratio of aligned residues (number of aligned residues divided by the length of ECOD domains) is not less than 95 %, and the number of corresponding residues is not less than 40.	31,470
train	The subset of the "all" set that was released before 2021-02-15.	29,274
test	The subset of the "all" set that was released after 2021-02-15.	2,196
noBLTtest	The subset of the "test" set containing proteins without homology detected by BLAST against the PDB proteins publicly released before 2021-02-10. The threshold *E*-value was 0.001.	350
noHHtest	The subset of the "test" set containing proteins without homology detected by BLAST and HHsearch against the PDB proteins publicly released before 2021-02-10. The threshold *E*-value was 0.001.	27

Table S2. Numbers of domains for different ranges of averaged pLDDT value.

**Range of residue-averaged pLDDT value**	**all set**	**test set**
30<pLDDT≤40	5	1
40<pLDDT≤50	11	3
50<pLDDT≤60	30	4
60<pLDDT≤70	158	12
70<pLDDT≤80	815	74
80<pLDDT≤90	5,552	561
90<pLDDT≤100	24,899	1,541
Total	31,470	2,196
