# Supplementary material for: Assessing Structural Classification Using AlphaFold2 Models Through ECOD‐Based Comparative Analysis
Source: Proteins. 2025 Apr 19;93(9):1571–85. doi: 10.1002/prot.26828 (PMC12314581; doi:10.1002/prot.26828)
Supplement: Supplementary file 2 — Data S2. [file PROT-93-1571-s002.pdf]

# Supplementary Figures

## Assessing Structural Classification Using AlphaFold2 Models through ECOD-based Comparative Analysis

Takeshi Kawabata, Kengo Kinoshita

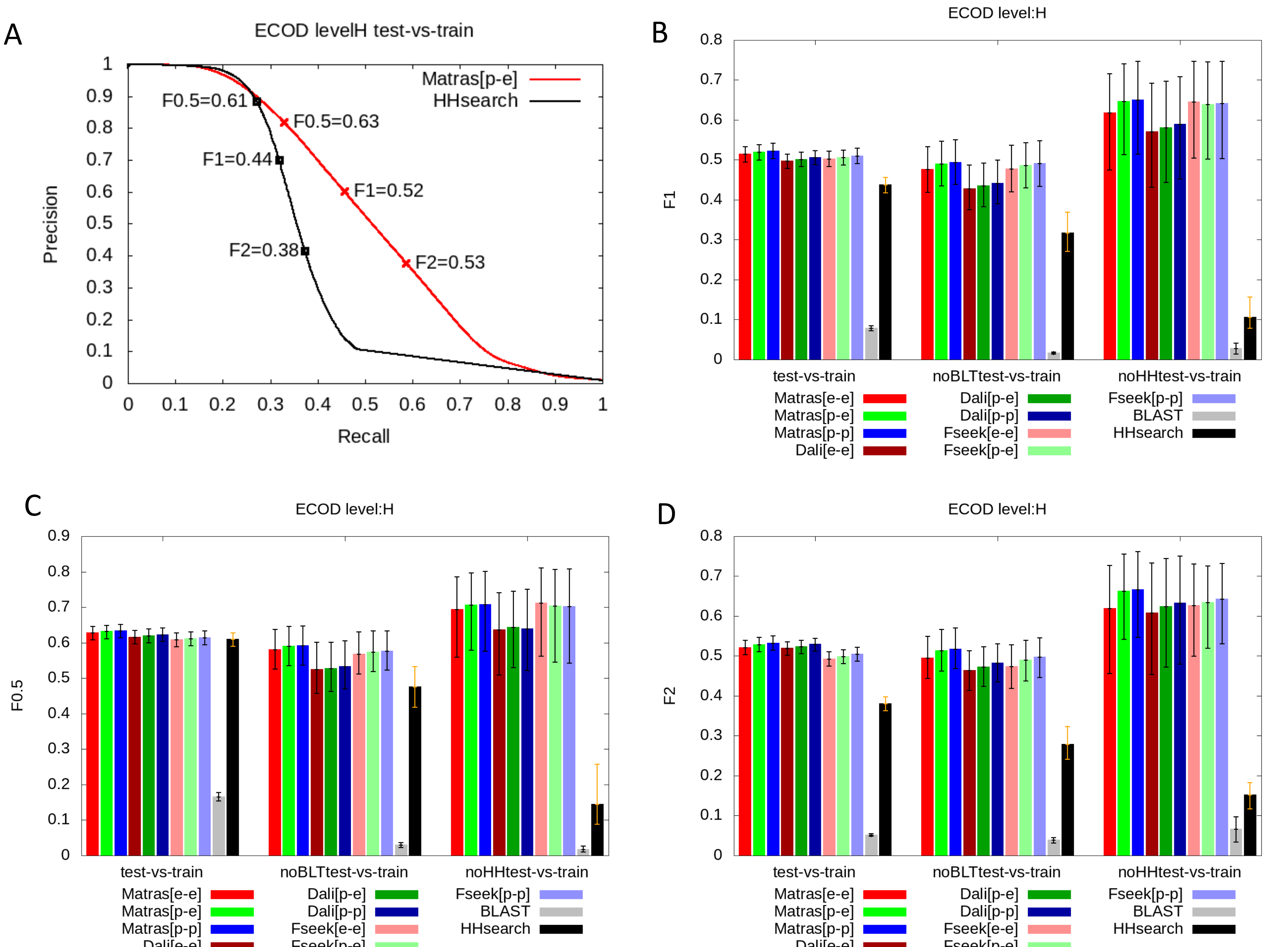

A
ECOD levelH test-vs-train
Matras[p-e]
HHsearch
F0.5=0.61
F0.5=0.63
F1=0.44
F1=0.52
F2=0.38
F2=0.53
Precision
Recall
B
ECOD level:H
F1
C
ECOD level:H
F0.5
D
ECOD level:H
F2
test-vs-train
noBLTtest-vs-train
noHHtest-vs-train
Matras[e-e]
Matras[p-e]
Matras[p-p]
Dali[e-e]
Dali[p-e]
Dali[p-p]
Fseek[e-e]
Fseek[p-e]
Fseek[p-p]
BLAST
HHsearch


Figure S1

**Figure S1.** Performances of various sequence and structure comparisons to discriminate between ECOD "H" levels and non-"H" levels for different combinations of the query and library sets. (A) A precision-recall plot of predicted structures in the "test" set versus experimental structures in the "train" set for Matras and HHsearch. Three $F$-scores ($F_{0.5}$, $F_1$, and $F_2$) are shown in the plot. (B) $F_1$-scores of various datasets, methods, and structures. "Fseek" stands for the program Foldseek. "[e-e]," "[p-e]," and "[p-p]" indicate comparisons between experimental and experimental structures, those between predicted and experimental structures, and those between predicted and predicted structures, respectively. (C) $F_{0.5}$-scores of various datasets, methods, and structures. (C) $F_{2.0}$-scores of multiple datasets, methods, and structures. The error bars in the bar graphs indicate the 95 % confidence intervals of $F_\beta$-scores estimated using the bootstrap method.

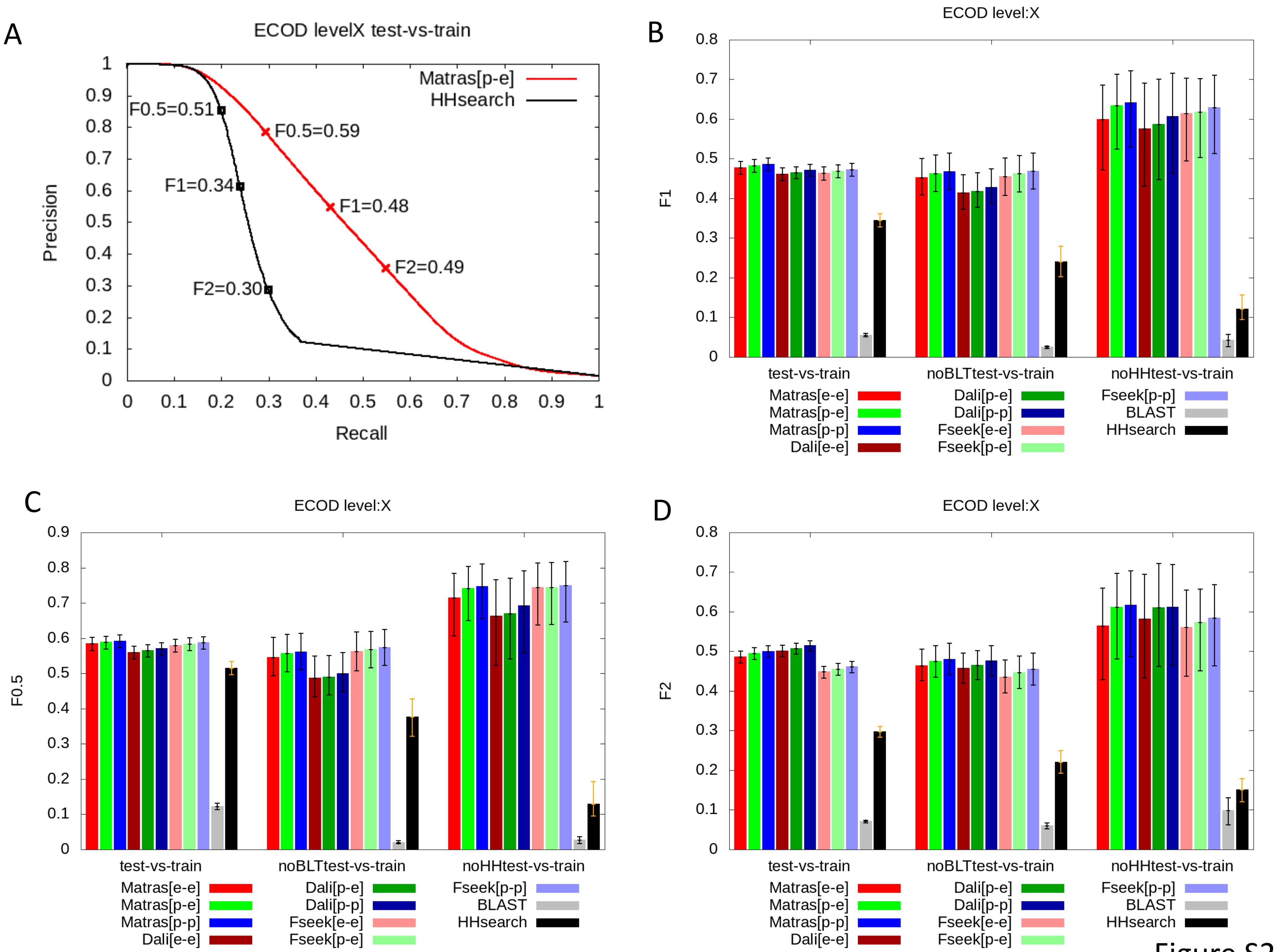
A
ECOD levelX test-vs-train
Matras[p-e]
HHsearch
F0.5=0.51
F0.5=0.59
F1=0.34
F1=0.48
F2=0.49
F2=0.30
Precision
Recall
B
ECOD level:X
F1
C
ECOD level:X
F0.5
D
ECOD level:X
F2
test-vs-train
noBLTtest-vs-train
noHHtest-vs-train
Matras[e-e]
Matras[p-e]
Matras[p-p]
Dali[e-e]
Dali[p-e]
Dali[p-p]
Fseek[e-e]
Fseek[p-e]
Fseek[p-p]
BLAST
HHsearch

Figure S2

**Figure S2.** Performances of various sequence and structure comparisons to discriminate ECOD “X” levels and non-”X” levels for different combinations of the query and library sets. (A) A precision-recall plot of predicted structures in the “test” set versus experimental structures in the “train” set for Matras and HHsearch. Three *F*-scores ($F_{0.5}$, $F_1$, and $F_2$) are shown in the plot. (B) $F_1$-scores of various datasets, methods, and structures. “Fseek” stands for the program Foldseek. “[e-e],” “[p-e],” and “[p-p]” indicate comparisons between experimental and experimental structures, those between predicted and experimental structures, and those between predicted and predicted structures, respectively. (C) $F_{0.5}$-scores of various datasets, methods, and structures. (C) $F_{2.0}$-scores of multiple datasets, methods, and structures. The error bars in the bar graphs indicate the 95 % confidence intervals of $F_\beta$- scores estimated using the bootstrap method.

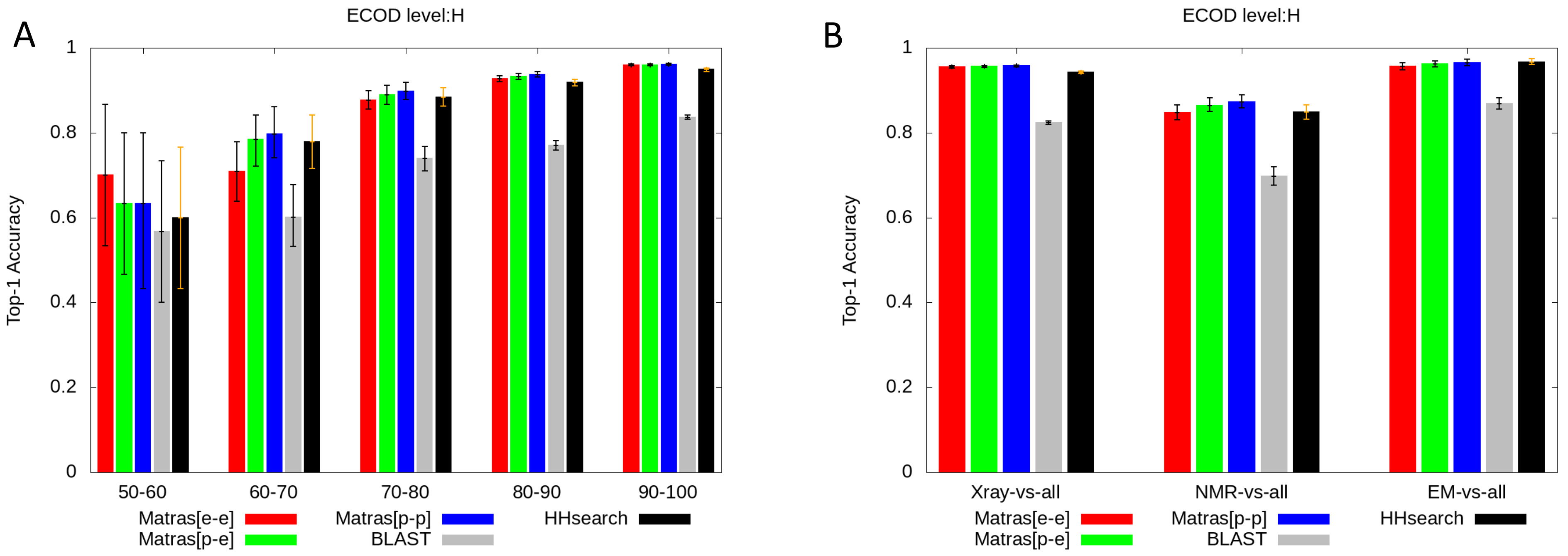


**Figure S3.** Performances of Matras, BLAST, and HHsearch to discriminate between ECOD "H" levels and non-"H" levels. (A) Top-1 accuracies of Matras, BLAST, and HHsearch for five different pLDDT value query sets against the "all" library set. (B) Top-1 accuracies of Matras, BLAST, and HHsearch for the three different sets of experimental methods vs the "all" dataset. The error bars in the bar graphs indicate the 95 % confidence intervals of top-1 accuracies estimated using the bootstrap method.

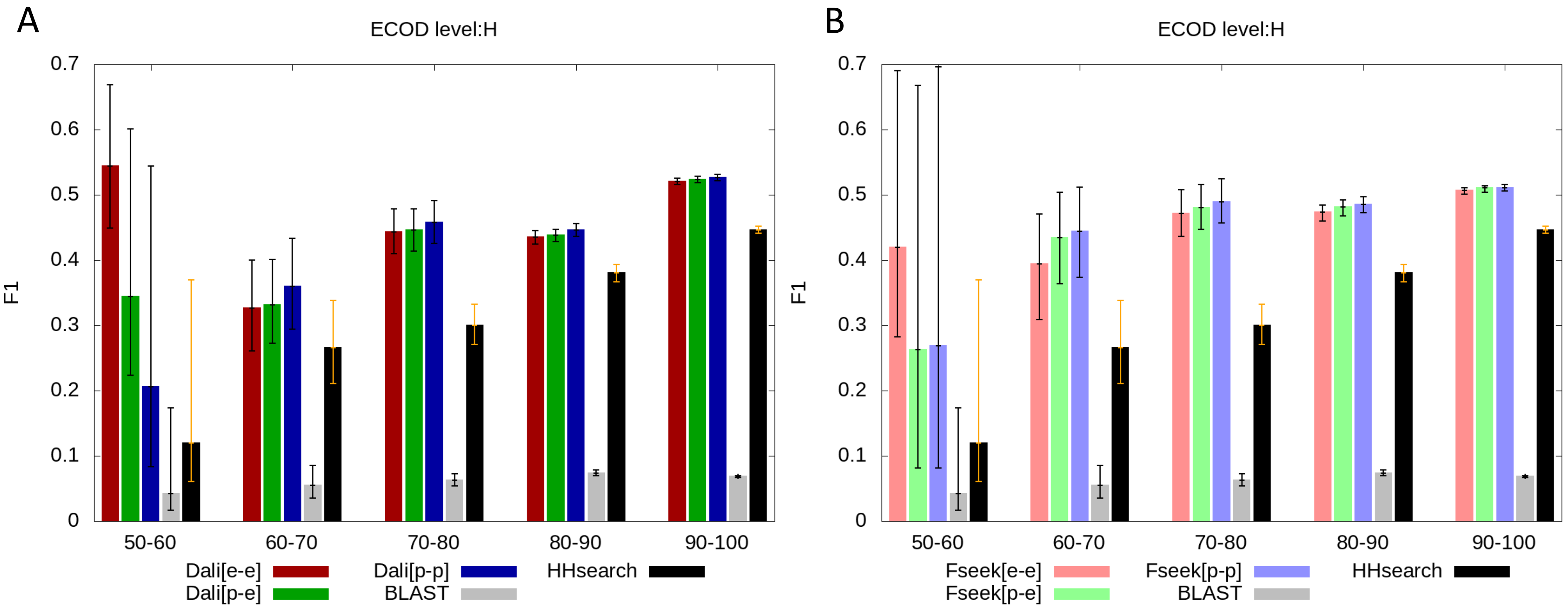


**Figure S4.** Performances of Dali, Foldseek, BLAST, and HHsearch to discriminate between ECOD "H" levels and non-"H" levels for five different pLDDT value query sets against the "all" library set. (A) $F_1$-scores of Dali, BLAST, and HHsearch. (B) $F_1$-scores of Foldseek, BLAST, and HHsearch. The error bars in the bar graphs indicate the 95 % confidence intervals of $F_1$-scores estimated using the bootstrap method. A corresponding graph for MATRAS is shown in Figure 5D.

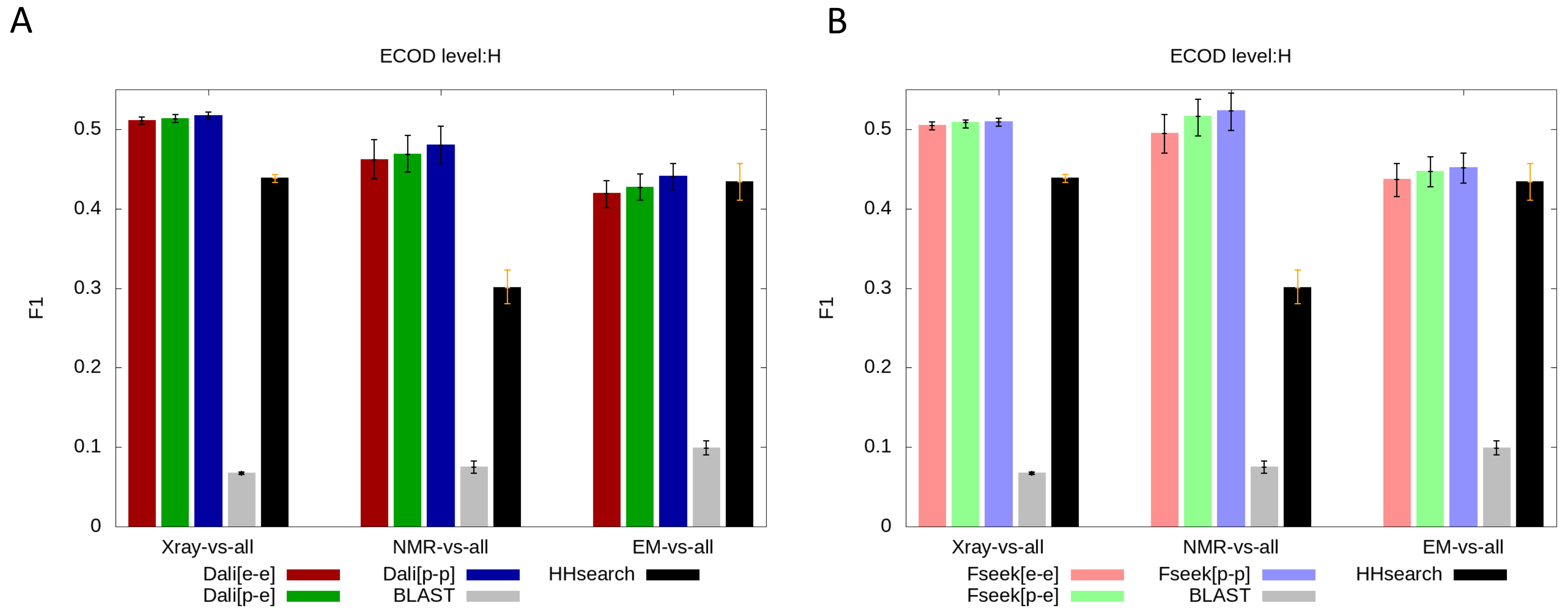


**Figure S5.** Performances of Dali, Foldseek, BLAST, and HHsearch to discriminate between ECOD "H" levels and non-"H" levels for the three different sets of experimental methods vs the "all" dataset. (A) $F_1$-scores of Dali, BLAST, and HHsearch. (B) $F_1$-scores of Foldseek, BLAST, and HHsearch. The error bars in the bar graphs indicate the 95 % confidence intervals of $F_1$-scores estimated using the bootstrap method. A corresponding graph for MATRAS is shown in Figure 7D.

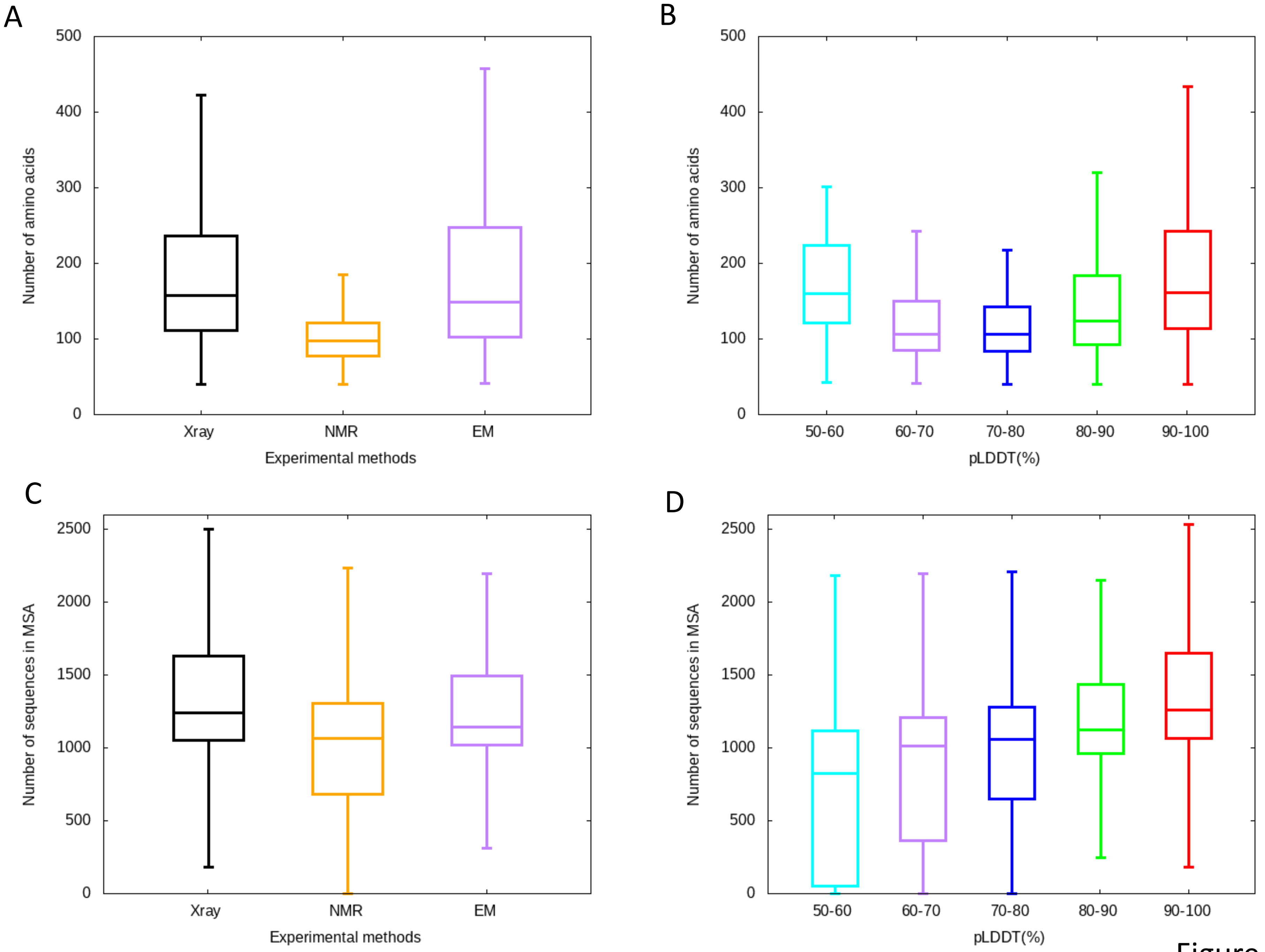
A
B
C
D
Number of amino acids
Experimental methods
Xray
NMR
EM
pLDDT(%)
50-60
60-70
70-80
80-90
90-100
Number of sequences in MSA
0
100
200
300
400
500
1000
1500
2000
2500

Figure S6

**Figure S6.** Distributions for number of amino acids and numbers of sequences in MSA. (A) A box plot of number of amino acids for three different experimental methods. (B) A box plot of number of amino acids for five different ranges of pLDDT values. (C) A box plot of number of sequences in MSA for three different experimental methods. (D) A box plot of number of sequences in MSA for five different ranges of pLDDT values.

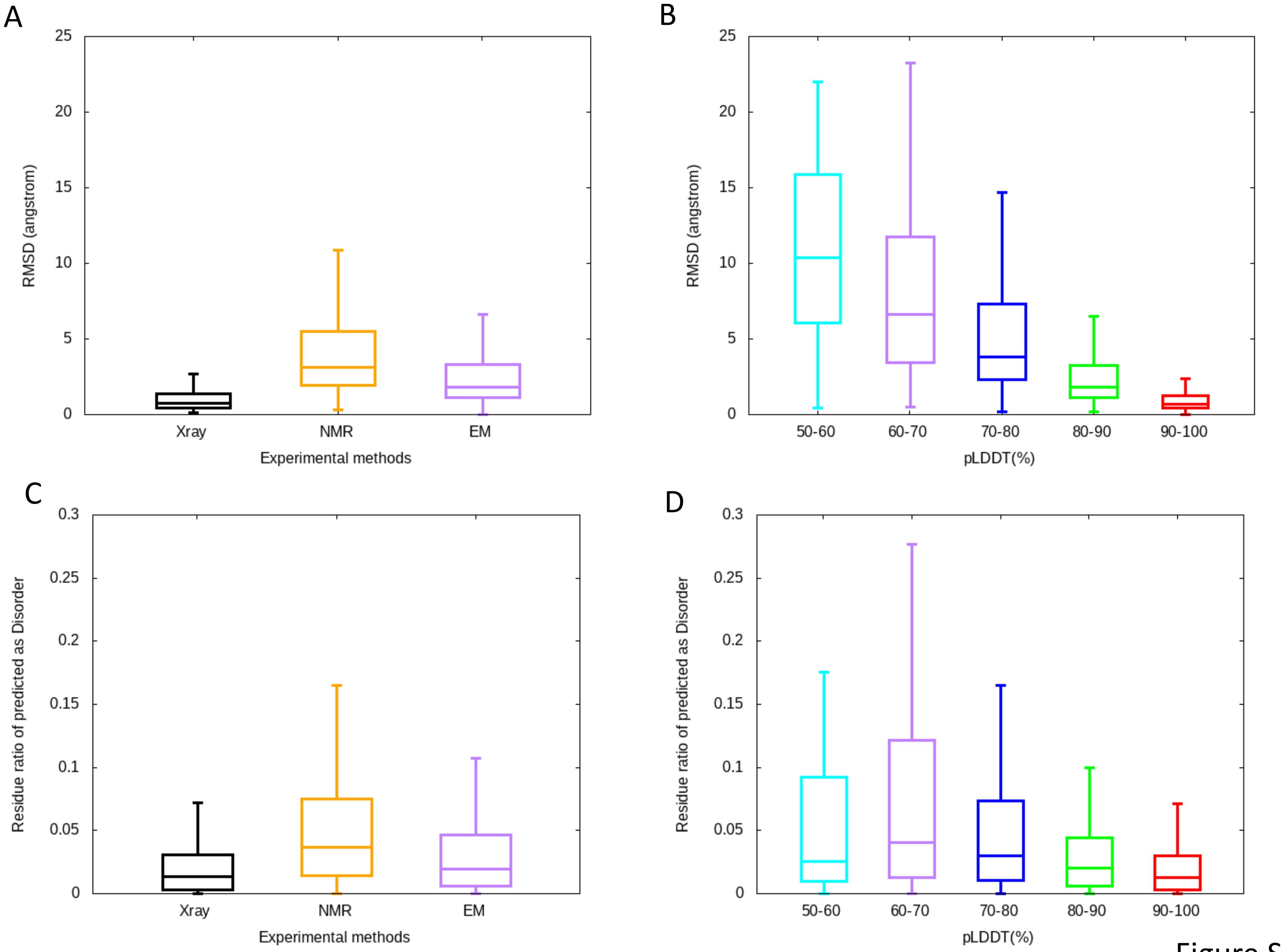

A
B
C
D
RMSD (angstrom)
Experimental methods
Xray
NMR
EM
pLDDT(%)
50-60
60-70
70-80
80-90
90-100
Residue ratio of predicted as Disorder
0
5
10
15
20
25
0.05
0.1
0.15
0.2
0.25
0.3


Figure S7

**Figure S7.** Distributions for RMSD between predicted and experimental structures and the ratio of residues predicted as disorder. (A) A box plot of RMSD for three different experimental methods. (B) A box plot of RMSD for five distinct ranges of pLDDT values. (C) A box plot of the ratio of residues predicted as a disorder for three different experimental methods. (D) A box plot of the ratio of residues predicted as a disorder for five distinct ranges of pLDDT values.

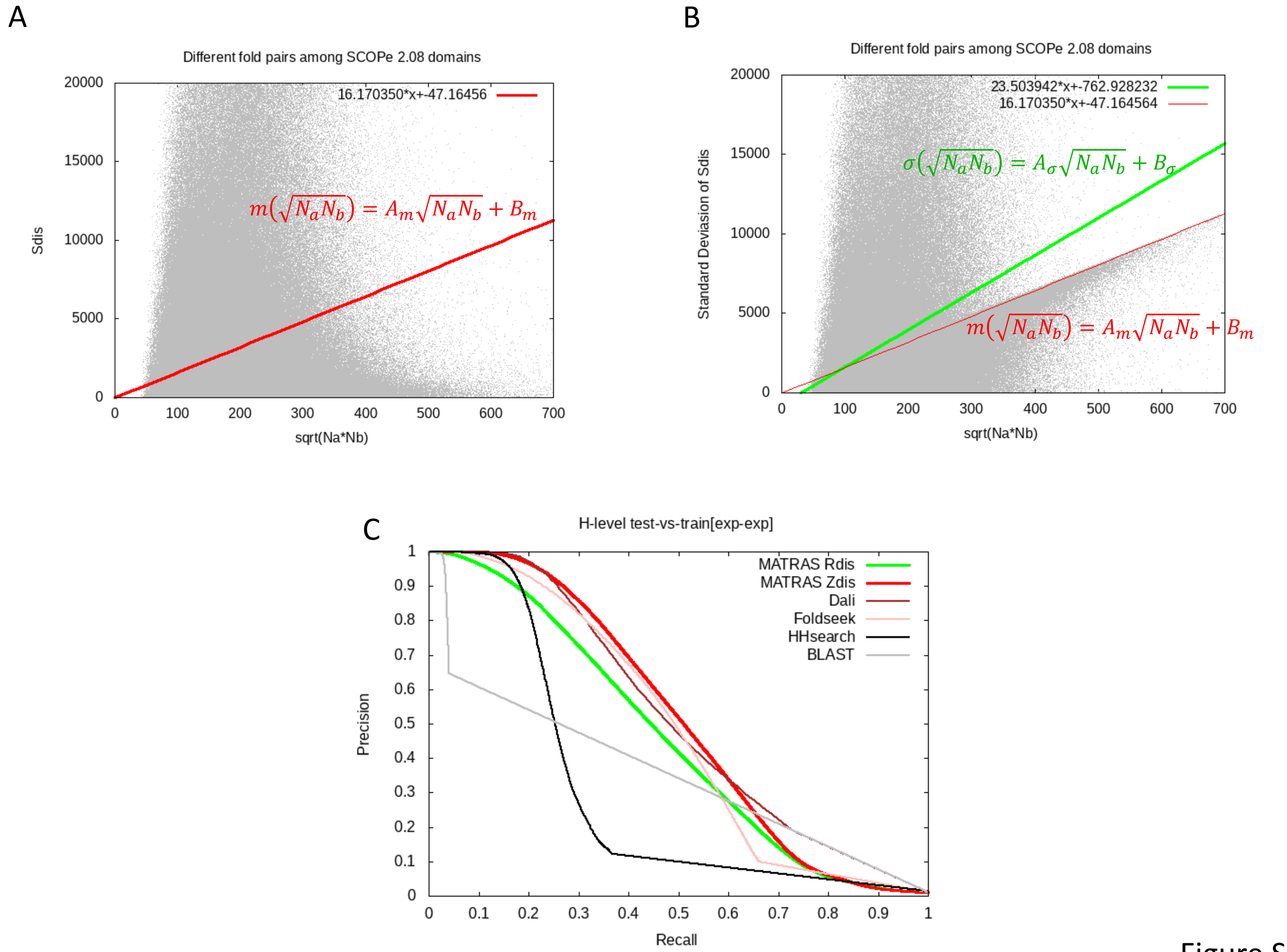
A
Different fold pairs among SCOPe 2.08 domains
16.170350*x+-47.16456
$m(\sqrt{N_a N_b}) = A_m\sqrt{N_a N_b} + B_m$
Sdis
sqrt(Na*Nb)
B
Different fold pairs among SCOPe 2.08 domains
23.503942*x+-762.928232
16.170350*x+-47.164564
$\sigma(\sqrt{N_a N_b}) = A_\sigma\sqrt{N_a N_b} + B_\sigma$
$m(\sqrt{N_a N_b}) = A_m\sqrt{N_a N_b} + B_m$
Standard Deviasion of Sdis
sqrt(Na*Nb)
C
H-level test-vs-train[exp-exp]
MATRAS Rdis
MATRAS Zdis
Dali
Foldseek
HHsearch
BLAST
Precision
Recall

Figure S8

**Figure S8.** Regression of MATRAS $S_{dis}$ score using geometric means of the length of compared proteins for normalizing to the $Z_{dis}$ score. The regression was performed using the 40% representative 14,225 domains of SCOPe 2.08. (A) $S_{dis}$ scores against geometric means of the length of compared proteins (gray dots) and the regression of the mean score $m$ (red line). (B) The standard deviations from the regressed mean score (gray dots) and the regression of the standard deviation $\sigma$ (green line) from the regressed mean score (red thin line). Clustered dots on the regressed mean score (red dotted line) indicate that many non-homologous structure pairs tend to have $S_{dis} = 0$ because MATRAS employs the Smith & Waterman algorithm. (C) A precision-recall plot to discriminate between ECOD "H" levels and non-"H" levels for the test-vs-train dataset using the experimental-vs-experimental structures. A red line corresponds to the comparison using $Z_{dis}$ score. A green line corresponds to the MATRAS comparison using $R_{dis}$ score, defined as $R_{dis} = 2S_{dis}(a,b)/[\, S_{dis}(a,a) + S_{dis}(b,b)]$.

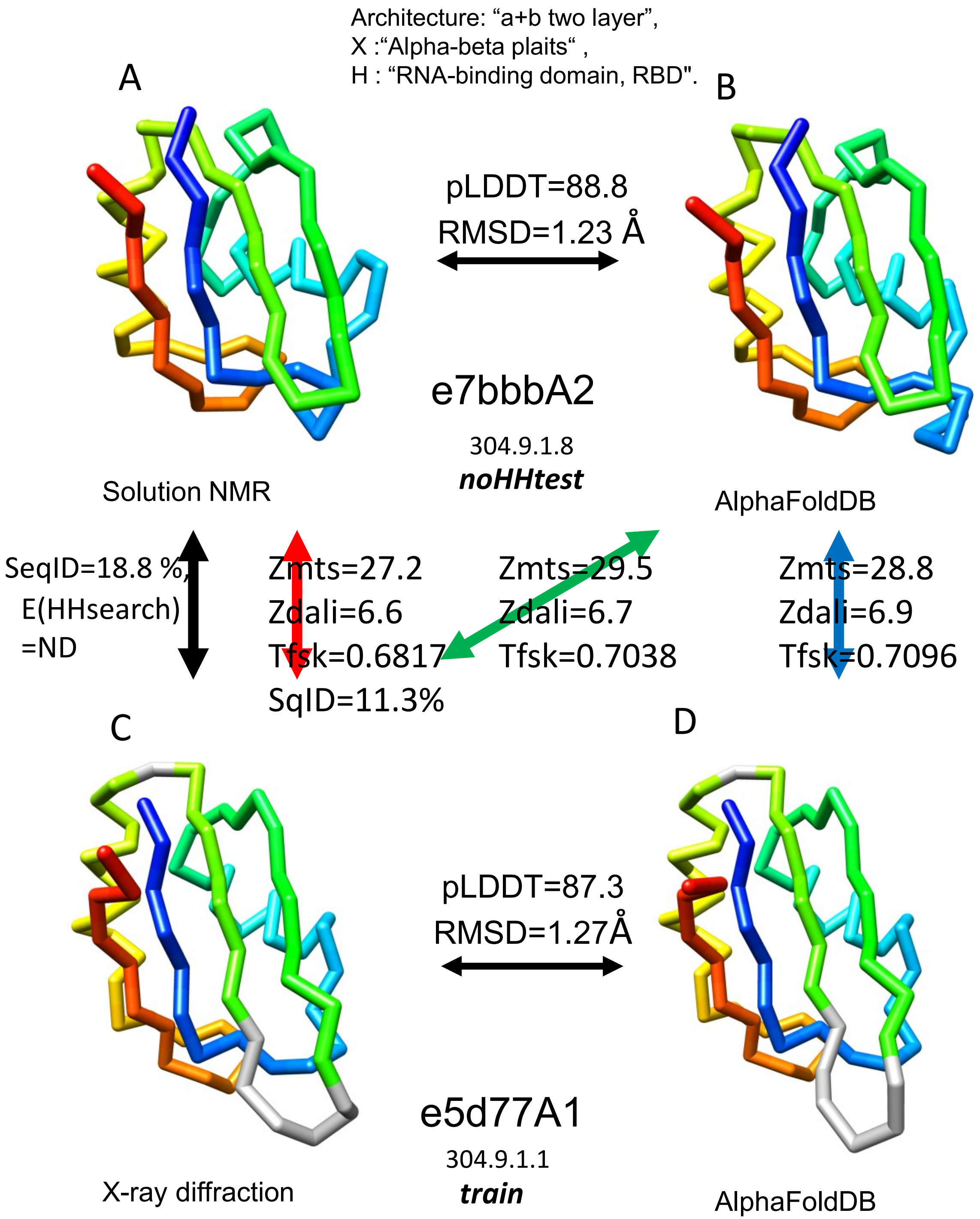

Architecture: “a+b two layer”,
X :“Alpha-beta plaits“ ,
H : “RNA-binding domain, RBD".
A
B
pLDDT=88.8
RMSD=1.23 Å
e7bbbA2
304.9.1.8
noHHtest
Solution NMR
AlphaFoldDB
SeqID=18.8 %,
E(HHsearch)
=ND
Zmts=27.2
Zdali=6.6
Tfsk=0.6817
SqID=11.3%
Zmts=29.5
Zdali=6.7
Tfsk=0.7038
Zmts=28.8
Zdali=6.9
Tfsk=0.7096
C
D
pLDDT=87.3
RMSD=1.27Å
e5d77A1
304.9.1.1
train
X-ray diffraction
AlphaFoldDB


Figure S9

**Figure S9.** A successful example where a structural comparison of a predicted structure recognized distant homology. In this case, the predicted structure shows performance comparable to experimental structures. HHsearch failed to recognize this homology. (A) An experimental structure and (B) a predicted structure for the query protein domain e7bbbA2. (C) An experimental structure and (D) A predicted structure for the library protein domain e5d77A1.

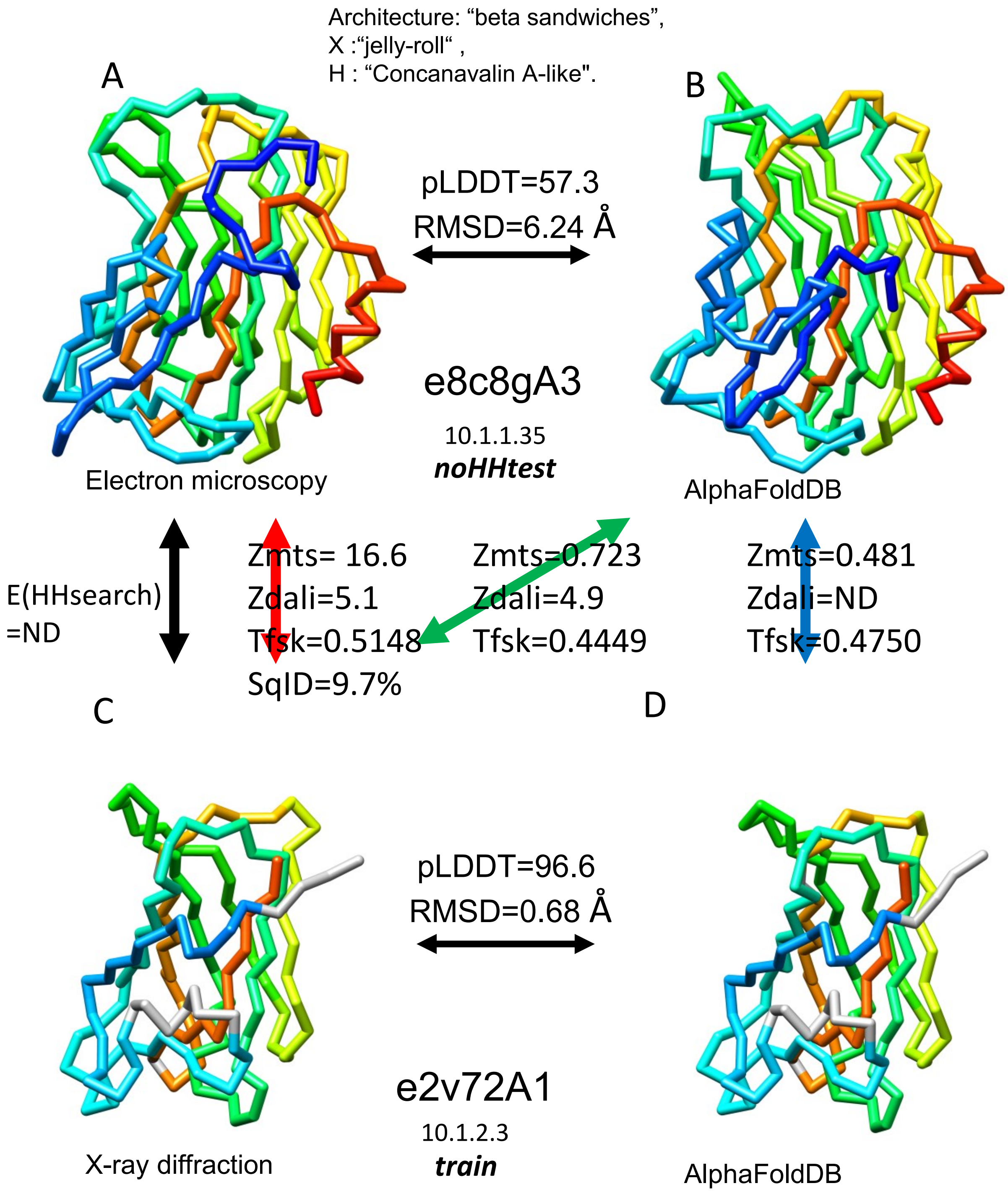
Architecture: “beta sandwiches”,
X :“jelly-roll“ ,
H : “Concanavalin A-like".
A
B
pLDDT=57.3
RMSD=6.24 Å
e8c8gA3
10.1.1.35
noHHtest
Electron microscopy
AlphaFoldDB
E(HHsearch)
=ND
Zmts= 16.6
Zdali=5.1
Tfsk=0.5148
SqID=9.7%
Zmts=0.723
Zdali=4.9
Tfsk=0.4449
Zmts=0.481
Zdali=ND
Tfsk=0.4750
C
D
pLDDT=96.6
RMSD=0.68 Å
e2v72A1
10.1.2.3
train
X-ray diffraction
AlphaFoldDB

Figure S10

**Figure S10.** An unsuccessful example where a structural comparison of a predicted structure failed to recognize distant homology. In this case, the experimental structure recognized a distant homology, whereas the predicted structure failed, probably because the accuracy of the query predicted structure was low. (A) An experimental structure and (B) a predicted structure for the query protein domain e8c8gA3. (C) An experimental structure and (D) A predicted structure for the library protein domain e2v72A1.

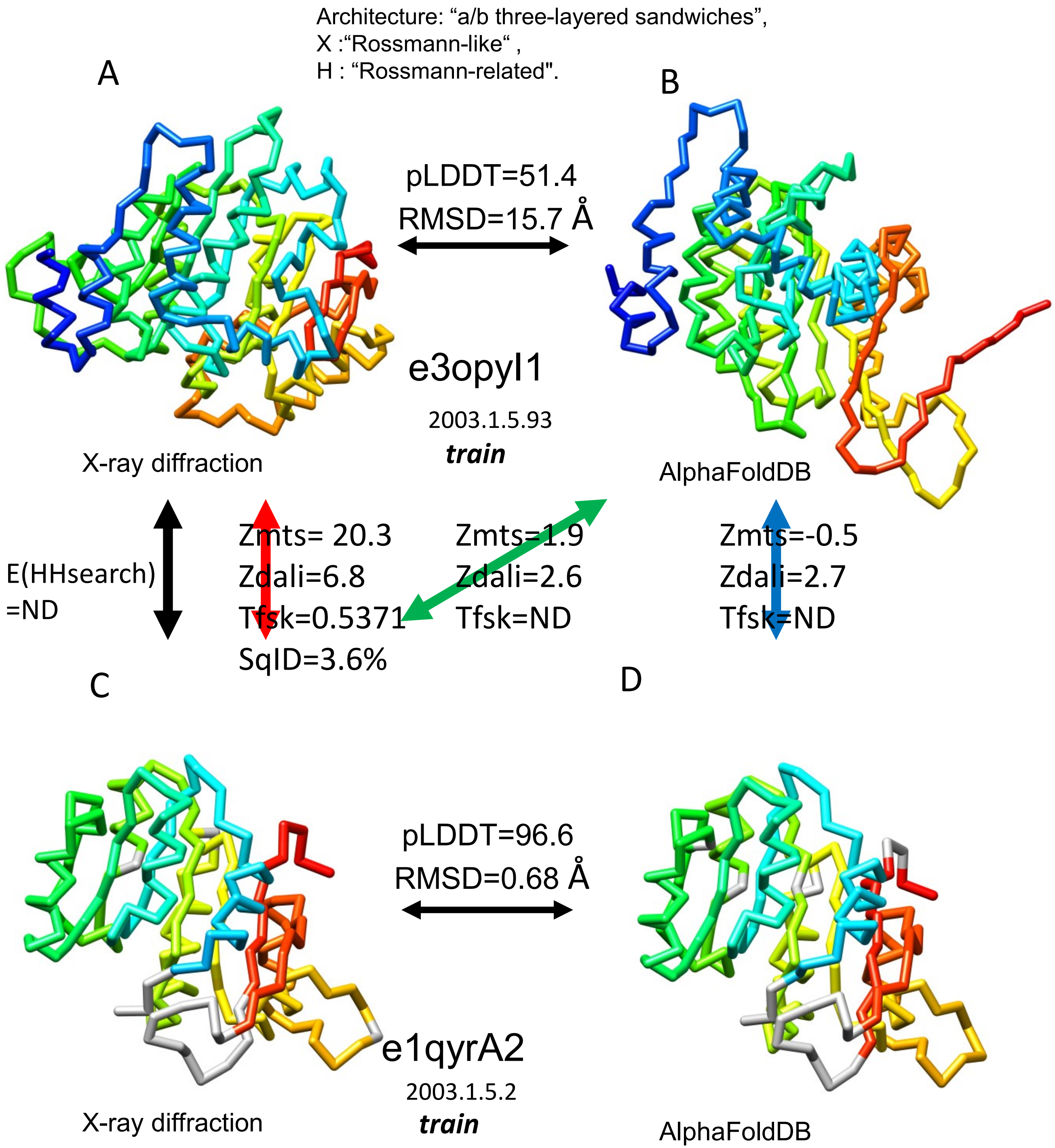
Architecture: "a/b three-layered sandwiches",
X :"Rossmann-like" ,
H : "Rossmann-related".
A
B
pLDDT=51.4
RMSD=15.7 Å
e3opyI1
2003.1.5.93
train
X-ray diffraction
AlphaFoldDB
E(HHsearch)
=ND
Zmts= 20.3
Zdali=6.8
Tfsk=0.5371
SqID=3.6%
Zmts=1.9
Zdali=2.6
Tfsk=ND
Zmts=-0.5
Zdali=2.7
Tfsk=ND
C
D
pLDDT=96.6
RMSD=0.68 Å
e1qyrA2
2003.1.5.2
train
X-ray diffraction
AlphaFoldDB

Figure S11

**Figure S11.** An unsuccessful example where a structural comparison of a predicted structure failed to recognize distant homology. In this case, the experimental structure recognized a distant homology, whereas the predicted structure failed, probably because the accuracy of the query predicted structure was low. (A) An experimental structure and (B) a predicted structure for the query protein domain e3opyI1. (C) An experimental structure and (D) A predicted structure for the library protein domain e1qyrA2.

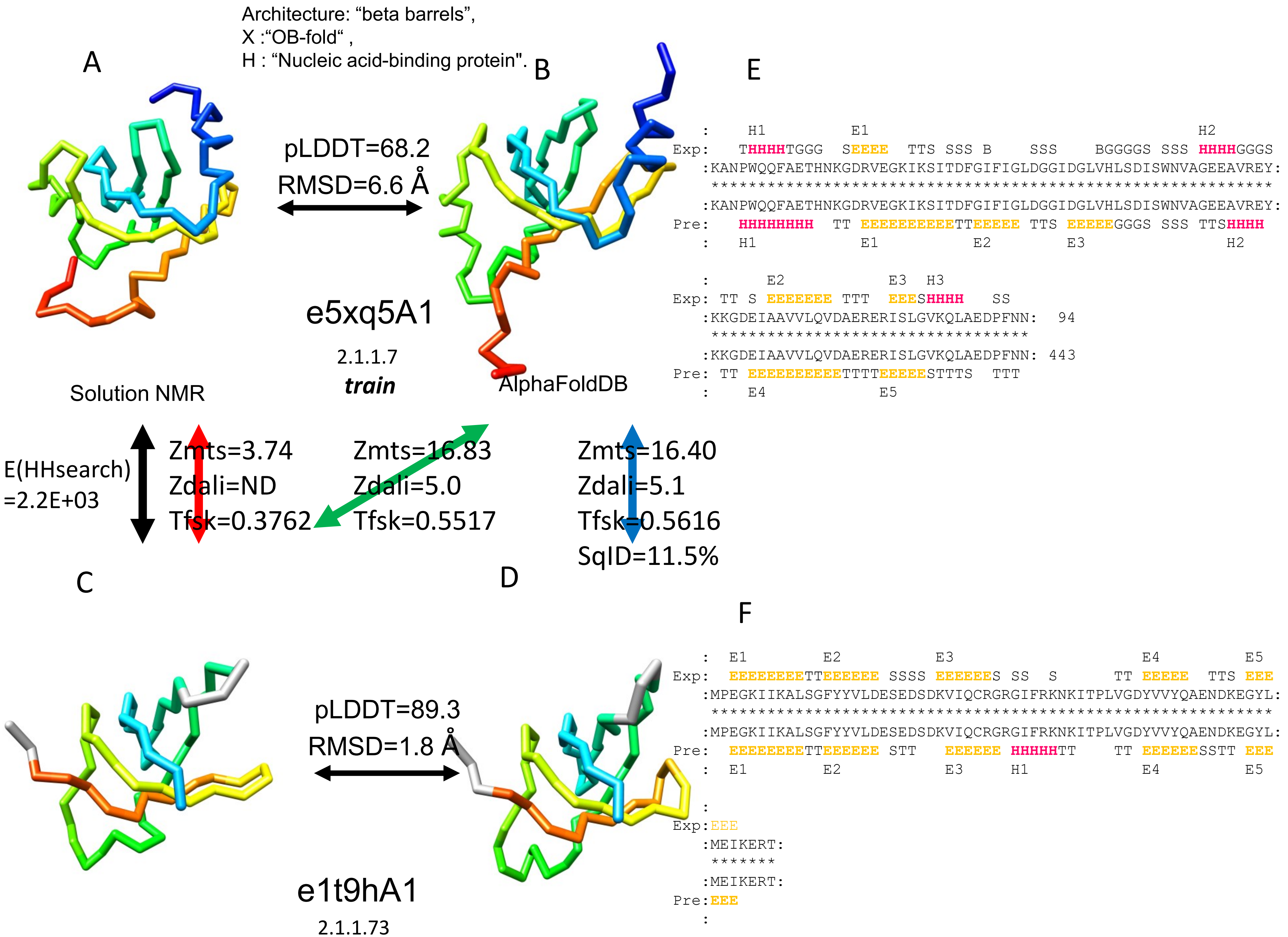

Architecture: “beta barrels”,
X :“OB-fold“ ,
H : “Nucleic acid-binding protein".
A
B
E
pLDDT=68.2
RMSD=6.6 Å
e5xq5A1
2.1.1.7
train
Solution NMR
AlphaFoldDB
: H1 E1 H2
Exp: THHHHTGGG SEEEE TTS SSS B SSS BGGGGS SSS HHHHGGGS
:KANPWQQFAETHNKGDRVEGKIKSITDFGIFIGLDGGIDGLVHLSDISWNVAGEEAVREY:
************************************************************
:KANPWQQFAETHNKGDRVEGKIKSITDFGIFIGLDGGIDGLVHLSDISWNVAGEEAVREY:
Pre: HHHHHHHH TT EEEEEEEEEETTEEEEE TTS EEEEEGGGS SSS TTSHHHH
: H1 E1 E2 E3 H2
: E2 E3 H3
Exp: TT S EEEEEEE TTT EEESHHHH SS
:KKGDEIAAVVLQVDAERERISLGVKQLAEDPFNN: 94
**********************************
:KKGDEIAAVVLQVDAERERISLGVKQLAEDPFNN: 443
Pre: TT EEEEEEEEEETTTTEEEEESTTTS TTT
: E4 E5
E(HHsearch)
=2.2E+03
Zmts=3.74
Zdali=ND
Tfsk=0.3762
Zmts=16.83
Zdali=5.0
Tfsk=0.5517
Zmts=16.40
Zdali=5.1
Tfsk=0.5616
SqID=11.5%
C
D
F
pLDDT=89.3
RMSD=1.8 Å
e1t9hA1
2.1.1.73
train
X-ray diffraction
AlphaFoldDB
: E1 E2 E3 E4 E5
Exp: EEEEEEEETTEEEEEE SSSS EEEEEESS SS S TT EEEEE TTS EEE
:MPEGKIIKALSGFYYVLDESEDSDKVIQCRGRGIFRKNKITPLVGDYVVYQAENDKEGYL:
************************************************************
:MPEGKIIKALSGFYYVLDESEDSDKVIQCRGRGIFRKNKITPLVGDYVVYQAENDKEGYL:
Pre: EEEEEEEETTEEEEEE STT EEEEEE HHHHHTT TT EEEEEESSTT EEE
: E1 E2 E3 H1 E4 E5
:
Exp:EEE
:MEIKERT:
*******
:MEIKERT:
Pre:EEE
:


Figure S12

**Figure S12.** An example where structural comparison using predicted structures better-recognized homology than the experimental structure. Solution NMR determined the query experimental structure. The predicted structure of the query contains more residues in α helices and β strands compared to the experimental structure. (A) An experimental structure and (B) A predicted structure for the query protein domain e5xq5A1. (C) An experimental structure and (D) A predicted structure for the library protein domain e1t9hA1. (E) An alignment between experimental and predicted structure for the query domain. (F) An alignment between experimental and predicted structure for the library domain.

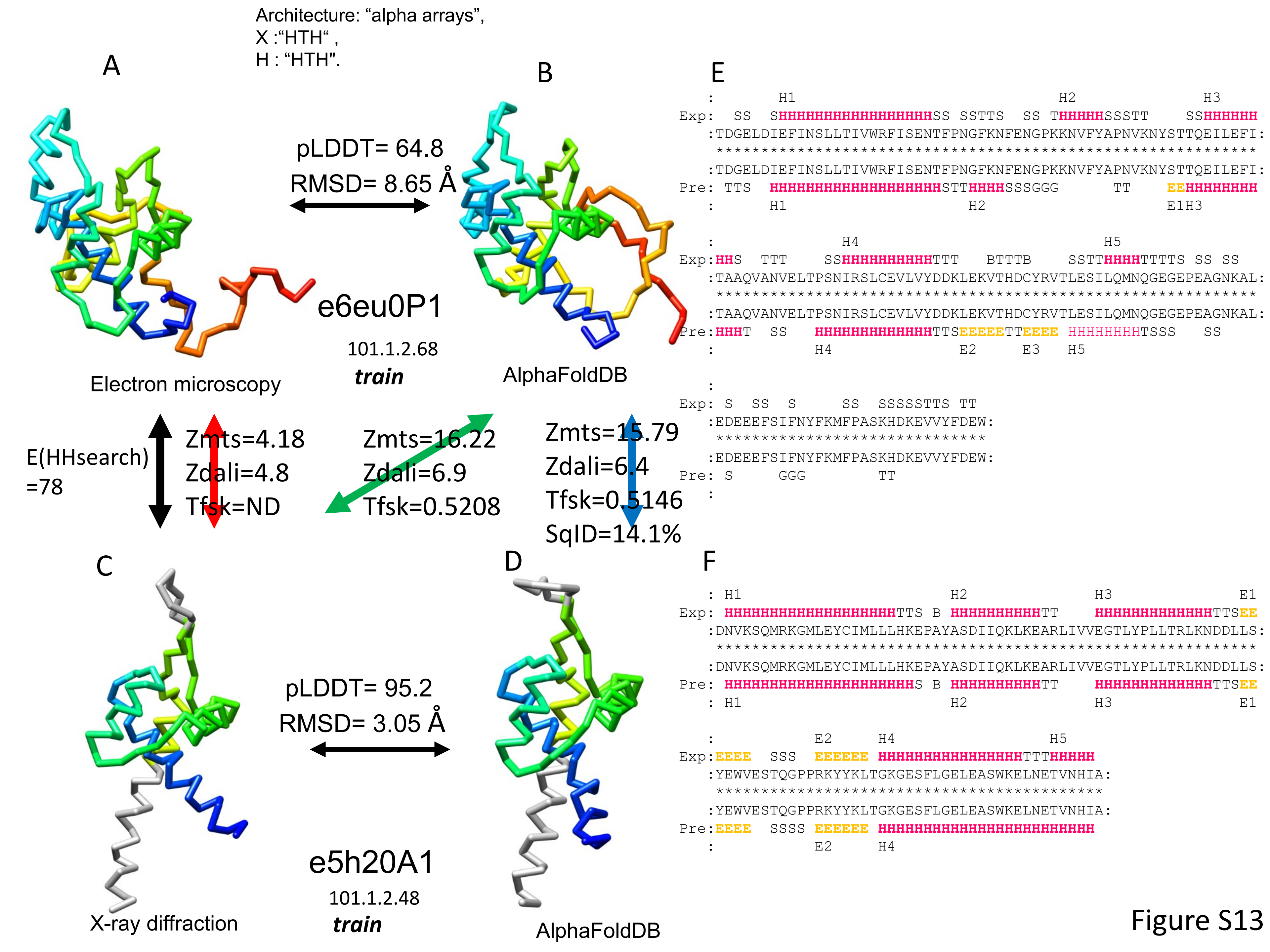

Architecture: “alpha arrays”,
X :“HTH“ ,
H : “HTH”.
A
B
pLDDT= 64.8
RMSD= 8.65 Å
e6eu0P1
101.1.2.68
train
Electron microscopy
AlphaFoldDB
E(HHsearch)
=78
Zmts=4.18
Zdali=4.8
Tfsk=ND
Zmts=16.22
Zdali=6.9
Tfsk=0.5208
Zmts=15.79
Zdali=6.4
Tfsk=0.5146
SqID=14.1%
C
D
pLDDT= 95.2
RMSD= 3.05 Å
e5h20A1
101.1.2.48
train
X-ray diffraction
AlphaFoldDB
E
   :       H1                             H2              H3
Exp:  SS  SHHHHHHHHHHHHHHHHHSS SSTTS  SS THHHHHSSSTT    SSHHHHHH
   :TDGELDIEFINSLLTIVWRFISENTFPNGFKNFENGPKKNVFYAPNVKNYSTTQEILEFI:
    ************************************************************
   :TDGELDIEFINSLLTIVWRFISENTFPNGFKNFENGPKKNVFYAPNVKNYSTTQEILEFI:
Pre: TTS  HHHHHHHHHHHHHHHHHHHSTTHHHHSSSGGG      TT    EEHHHHHHHH
   :      H1                    H2                    E1H3
   :              H4                           H5
Exp:HHS  TTT    SSHHHHHHHHHHTTT   BTTTB    SSTTHHHHTTTTS SS SS
   :TAAQVANVELTPSNIRSLCEVLVYDDKLEKVTHDCYRVTLESILQMNQGEGEPEAGNKAL:
    ************************************************************
   :TAAQVANVELTPSNIRSLCEVLVYDDKLEKVTHDCYRVTLESILQMNQGEGEPEAGNKAL:
Pre:HHHT  SS   HHHHHHHHHHHHHTTSEEEEETTEEEE HHHHHHHHTSSS   SS
   :           H4              E2     E3   H5
   :
Exp: S  SS  S     SS  SSSSSTTS TT
   :EDEEEFSIFNYFKMFPASKHDKEVVYFDEW:
    ******************************
   :EDEEEFSIFNYFKMFPASKHDKEVVYFDEW:
Pre: S     GGG        TT
   :
F
   : H1                       H2              H3              E1
Exp: HHHHHHHHHHHHHHHHHHHTTS B HHHHHHHHHHTT    HHHHHHHHHHHHHTTSEE
   :DNVKSQMRKGMLEYCIMLLLHKEPAYASDIIQKLKEARLIVVEGTLYPLLTRLKNDDLLS:
    ************************************************************
   :DNVKSQMRKGMLEYCIMLLLHKEPAYASDIIQKLKEARLIVVEGTLYPLLTRLKNDDLLS:
Pre: HHHHHHHHHHHHHHHHHHHHHS B HHHHHHHHHHTT    HHHHHHHHHHHHHTTSEE
   : H1                       H2              H3              E1
   :           E2     H4                 H5
Exp:EEEE  SSS  EEEEEE HHHHHHHHHHHHHHHHTTTHHHHH
   :YEWVESTQGPPRKYYKLTGKGESFLGELEASWKELNETVNHIA:
    *******************************************
   :YEWVESTQGPPRKYYKLTGKGESFLGELEASWKELNETVNHIA:
Pre:EEEE  SSSS EEEEEE HHHHHHHHHHHHHHHHHHHHHHHH
   :           E2     H4


Figure S13

**Figure S13.** An example where structural comparison using predicted structures better-recognized homology than the experimental structure. Electron microscopy determined the query experimental structure. The predicted structure of the query contains more residues in α helices and β strands compared to the experimental structure. (A) An experimental structure and (B) a predicted structure for the query protein domain e6eu0P1. (C) An experimental structure and (D) A predicted structure for the library protein domain e5h20A1. (E) An alignment between experimental and predicted structure for the query domain. (F) An alignment between experimental and predicted structure for the library domain.

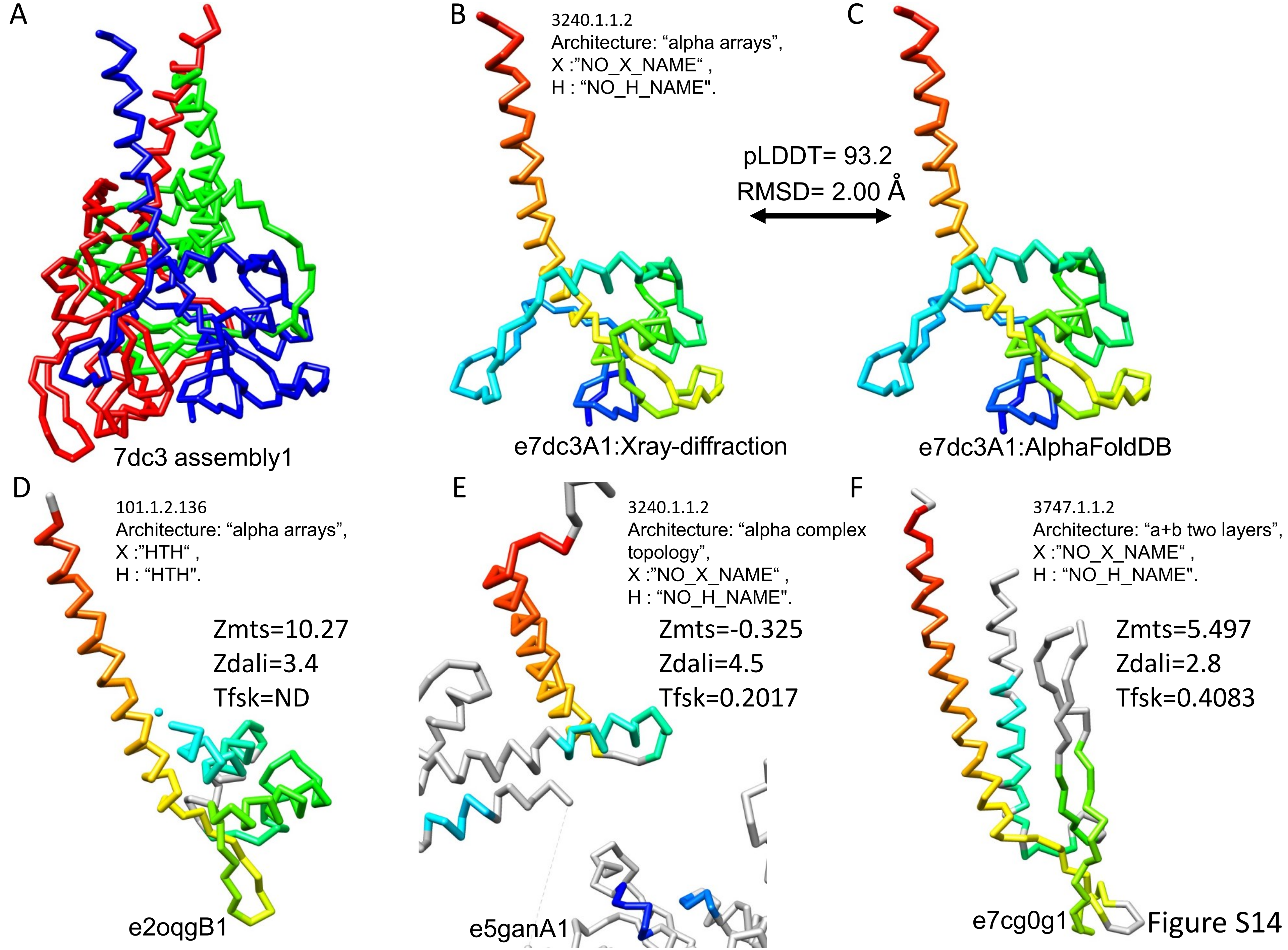
A
7dc3 assembly1
B
3240.1.1.2
Architecture: "alpha arrays",
X :"NO_X_NAME" ,
H : "NO_H_NAME".
e7dc3A1:Xray-diffraction
pLDDT= 93.2
RMSD= 2.00 Å
C
e7dc3A1:AlphaFoldDB
D
101.1.2.136
Architecture: "alpha arrays",
X :"HTH" ,
H : "HTH".
Zmts=10.27
Zdali=3.4
Tfsk=ND
e2oqgB1
E
3240.1.1.2
Architecture: "alpha complex topology",
X :"NO_X_NAME" ,
H : "NO_H_NAME".
Zmts=-0.325
Zdali=4.5
Tfsk=0.2017
e5ganA1
F
3747.1.1.2
Architecture: "a+b two layers",
X :"NO_X_NAME" ,
H : "NO_H_NAME".
Zmts=5.497
Zdali=2.8
Tfsk=0.4083
e7cg0g1
Figure S14

G

| #[method:1] | [strpair:2] | [setpair:3] | [ECOD:4] | [Condition:5] | [Sthre:6] | [Evalue:7] | [F05:8] | [F1:9] | [F2:10] | [recall:11] | [precision:12] |
|---|---|---|---|---|---|---|---|---|---|---|---|
| Matras_ZsqAB | pre-exp | test-vs-train | X | pre099 | **23.63** | - | 0.413486 | 0.220702 | 0.150523 | 0.124195 | 0.98999 |
| Matras_ZsqAB | pre-exp | test-vs-train | X | pre09 | **17.05** | - | 0.556405 | 0.353764 | 0.25932 | 0.22014 | 0.900152 |
| Matras_ZsqAB | pre-exp | test-vs-train | X | F05 | **13.9** | - | 0.588044 | 0.426123 | 0.334121 | 0.29208 | 0.787551 |
| Matras_ZsqAB | pre-exp | test-vs-train | X | F1 | **9.39** | - | 0.520472 | 0.482362 | 0.449452 | 0.429899 | 0.54941 |
| Matras_ZsqAB | pre-exp | test-vs-train | X | F2 | **5.97** | - | 0.382817 | 0.431601 | 0.494634 | 0.547987 | 0.355992 |
| Dali_Zscore | pre-exp | test-vs-train | X | pre099 | **8.8** | - | 0.425444 | 0.229251 | 0.156898 | 0.129624 | 0.990634 |
| Dali_Zscore | pre-exp | test-vs-train | X | pre09 | **6.6** | - | 0.536959 | 0.334516 | 0.242928 | 0.205431 | 0.900116 |
| Dali_Zscore | pre-exp | test-vs-train | X | F05 | **5.4** | - | 0.564195 | 0.405284 | 0.316218 | 0.27581 | 0.76387 |
| Dali_Zscore | pre-exp | test-vs-train | X | F1 | **3.8** | - | 0.483882 | 0.464624 | 0.44684 | 0.435721 | 0.497633 |
| Dali_Zscore | pre-exp | test-vs-train | X | F2 | **2.6** | - | 0.333605 | 0.402551 | 0.507419 | 0.614065 | 0.299417 |
| Foldseek_TMscore | pre-exp | test-vs-train | X | pre099 | **0.59** | - | 0.333547 | 0.16722 | 0.111579 | 0.091322 | 0.990067 |
| Foldseek_TMscore | pre-exp | test-vs-train | X | pre09 | **0.52** | - | 0.535888 | 0.333277 | 0.241841 | 0.204447 | 0.901092 |
| Foldseek_TMscore | pre-exp | test-vs-train | X | F05 | **0.47** | - | 0.582613 | 0.424567 | 0.33397 | 0.292377 | 0.774926 |
| Foldseek_TMscore | pre-exp | test-vs-train | X | F1 | **0.42** | - | 0.527744 | 0.468151 | 0.420651 | 0.393999 | 0.576684 |
| Foldseek_TMscore | pre-exp | test-vs-train | X | F2 | **0.36** | - | 0.39132 | 0.420824 | 0.45514 | 0.481305 | 0.373847 |

**Figure S14.** An example of "new group" in our dataset. A structure of the Myelin regulatory factor(MyRF) intramolecular chaperone autocleavage (ICA) domain (PDB_ID: 7dc3). (A) Experimental homo trimer structure for assembly 1 of PDB_ID: 7dc3. (B) a experimental structure for the query protein domain e7dc3A1. (C) A predicted structure for e7dc3A1. (D) Most similar structure e2oqgB1 for the predicted structure e7dc3A1 using Matras. (E) Most similar structure e5ganA1 for the predicted structure e7dc3A1 using Dali. (F) Most similar structure e7cg0g1 for the predicted structure e7dc3A1 using Foldseek. (G) The score thresholds for Matras, Dali and Foldseek for predicted vs. experimental structure pairs using test-vs-train datasets for "X" level. The similarity scores shown in Figure S14DEF were below the threshold for a precision of 0.9. The score thresholds for other methods and datasets can be downloaded from the online repository ( https://doi.org/10.5281/zenodo.14523133 ).
